# Exercise prescription for mood and cognition: targeting the microbiota-gut-brain axis through short-chain fatty acids

**DOI:** 10.3389/fmicb.2026.1740680

**Published:** 2026-04-13

**Authors:** Jin Xie, Junfeng Zhang, Lulu Zhang, Xiaoyan Chen

**Affiliations:** 1 Hubei University of Chinese Medicine, Wuhan, China; 2 Department of Rehabilitation, Taihe Hospital, Hubei University of Medicine, Shiyan, China; 3 Department of Hematology, Taihe Hospital, Hubei University of Medicine, Shiyan, China; 4 School of Basic Medical Sciences, Hubei University of Chinese Medicine, Wuhan, China

**Keywords:** dietary fiber, epigenetics, exercise, gut microbiota, immunity and barrier, microbiota-gut-brain axis, precision exercise, short chain fatty acids

## Abstract

Scientific study has extensively corroborated the advantageous impacts of exercise on mood, cognitive function, and stress resilience. Nonetheless, the fundamental biological mechanisms underpinning these effects have yet to be thoroughly integrated. This review advocates for and substantiates an integrated model focused on the “Exercise-Gut Microbiome-Short-Chain Fatty Acids (SCFAs)-Brain Function” axis. Consistent physical exercise alters the gut microbiota, enhancing Short-Chain Fatty Acid (SCFA)-producing populations, which is associated with markedly elevated bioavailability of key metabolites (acetate, propionate, and butyrate). Rather than detailing exhaustive molecular pathways here, we emphasize that these SCFAs facilitate gut-brain communication through multiple synergistic routes, including receptor-mediated neuroendocrine signaling, epigenetic modulation of neuroplasticity, and the attenuation of systemic neuroinflammation. Current human observational and interventional data strongly support an associative link between exercise-induced SCFA fluctuations and improved mental health outcomes. Crucially, we propose the novel “Exercise × Fiber Synergy” hypothesis: exercise primes the intestinal ecological niche for efficient substrate-utilizing bacteria, while adequate fermentable dietary fiber provides the necessary raw materials. Synergistically, this combination optimizes SCFA production to maximize cognitive and emotional benefits. To transition this framework into clinical practice, future research must prioritize 2 × 2 factorial designs (Exercise × Fiber) with dynamic kinetic measurements, paving the way for microbial phenotype-oriented precision exercise and personalized nutritional interventions to enhance public mental health.

## 1 Introduction

The worldwide prevalence of mental health issues is increasing, with depression, anxiety, and associated cognitive deficits exerting enduring effects on personal wellbeing, familial dynamics, and societal productivity (Covid-19 Mental Disorders Collaborators, 2021; GBD 2019 Mental Disorders Collaborators, 2022; Vigo et al., 2016). While current medications and psychotherapy benefit certain individuals, issues such as side effects, delayed efficacy, limited response rates, high relapse rates, and practical obstacles like resource accessibility and adherence underscore the pressing necessity for safe, scalable, cost-effective, and complementary lifestyle interventions (Cipriani et al., 2018). A multitude of epidemiological studies and randomized controlled trials have demonstrated that regular exercise can markedly mitigate symptoms of depression and anxiety (Novak and Goolsby, 2023; Singh et al., 2023), enhance executive function, memory, and learning capacity (Northey et al., 2018), and bolster stress resilience (Childs and de Wit, 2014); its advantages transcend age and gender, providing both physical and psychological benefits. Nonetheless, a significant obstacle persists: despite the evident benefits of exercise, there is an absence of a cohesive and verifiable biological mechanism framework, which hinders the advancement and practical use of “precision exercise prescriptions” (McGreevy et al., 2019).

**FIGURE 1:**
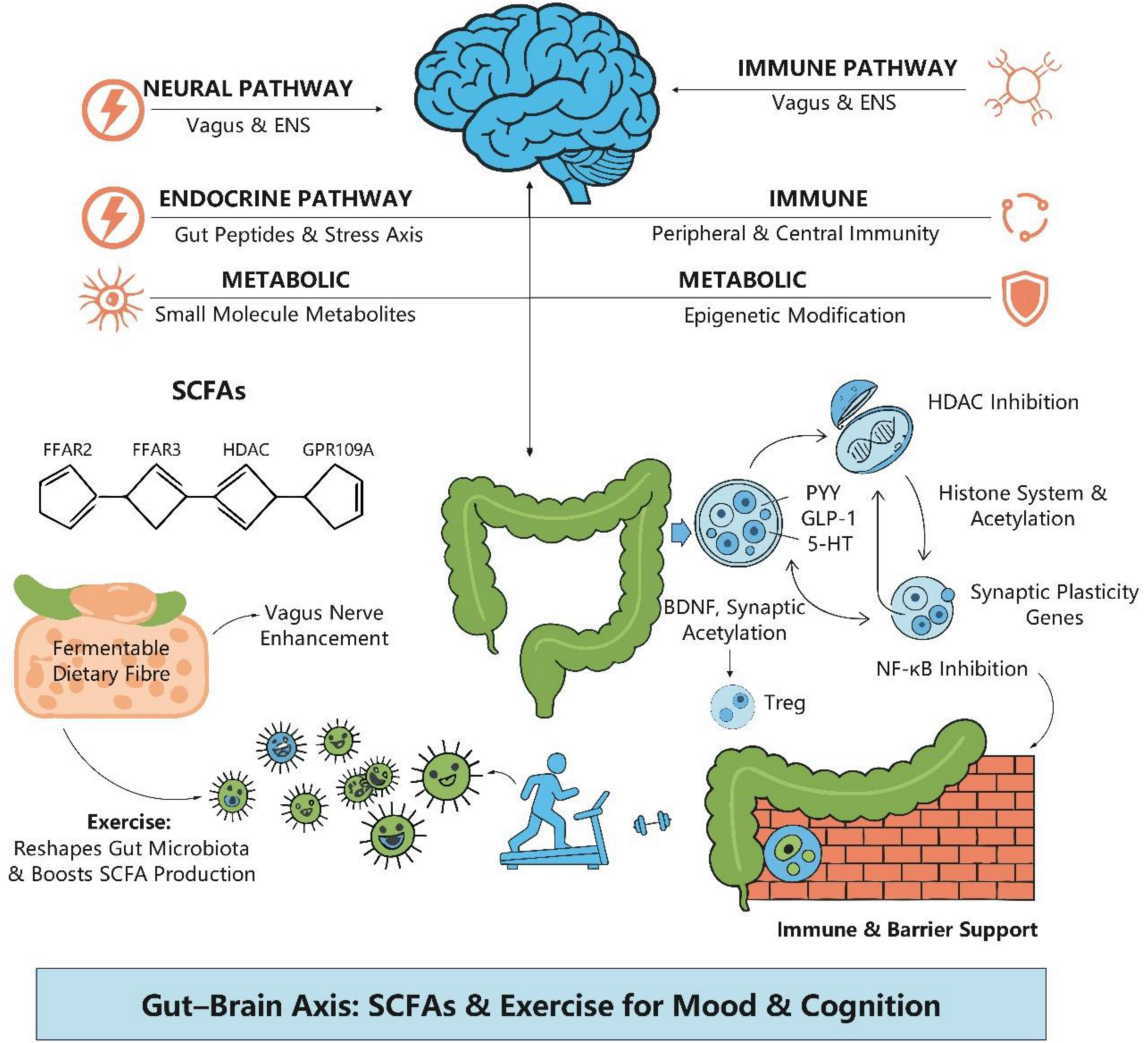
Exercise improves mood and cognition through the microbiota-gut-brain axis, with SCFAs playing a core role. As a positive lifestyle intervention, exercise reshapes the composition and metabolic function of the gut microbiota, promoting the proliferation of SCFA-producing taxa. The resulting elevation in colonic SCFAs triggers a multi-systemic cascade. SCFAs act locally to fortify the intestinal barrier, communicate via the enteric nervous system, and enter the systemic circulation to modulate peripheral immunity and directly influence central nervous system health, ultimately enhancing stress resilience and cognitive performance. Figure was created with EdrawMax.

The swiftly evolving “microbiota-gut-brain axis” in recent years has established a robust framework for synthesizing the psycho-physiological impacts of exercise (Cryan et al., 2020; Morais et al., 2021). The gut microbiota engages in bidirectional communication with the brain via various pathways: neurological, endocrine, immunological, and metabolic (Dalile et al., 2019; Osadchiy et al., 2019). Short-Chain Fatty Acids (SCFAs), as pivotal signaling molecules generated through the fermentation of fermentable dietary fibers by microbes, exert a cohesive influence across the gut-systemic-central axis (Silva et al., 2020). Initially, SCFAs modulate the production of PYY, GLP-1, and 5-HT by enteroendocrine cells and augment vagal afferent transmission via receptors including FFAR2, FFAR3, and GPR109A (Di Nunzio, 2023; Petropoulos et al., 2025). Secondly, butyrate and other compounds exhibit epigenetic action by inhibiting HDAC and facilitating various histone acetylation changes, thereby enhancing the production of BDNF and genes associated with neural plasticity (Fung et al., 2017). Thirdly, SCFAs are implicated in enhancing cerebral health by fortifying the intestinal barrier, promoting Treg induction, and suppressing the NF-κB pathway, thus diminishing systemic and neuroinflammation (Parker et al., 2020; Parada Venegas et al., 2019a). SCFAs are mostly digested in the colon, with the residual quantity being conveyed into the portal vein and systemic circulation via MCT1 and SMCT1 transmembrane transporters, establishing a foundation for their systemic signaling effects (Dirks et al., 2025). Current consensus indicates that exercise can modify the gut microbiome and increase the synthesis of SCFAs (Mailing et al., 2019). This leads to the formulation of a compelling and testable hypothesis: exercise exerts its beneficial effects on mood and cognition, in part, through the modulation of the microbiota’s composition and metabolic function. This modulation is associated with the augmented production and flow of SCFAs, potentially influencing the central network via many pathways, including receptor signaling, epigenetics, immunological barriers, and the vagus nerve (Figure 1; Cataldi et al., 2022). This review seeks to systematically consolidate animal and human evidence to evaluate the proposed pathway “exercise → microbiota alteration → enhanced SCFAs → multi-mechanism mediation → improved mood/cognition,” underscore the pivotal regulatory and amplifying function of dietary fiber in this process (Petropoulos et al., 2025), and propose a research agenda that integrates precision exercise and personalized nutrition informed by microbial phenotypes to promote public mental health and social cohesion (Kirk et al., 2021).

This review employs a hybrid approach of “narrative review combined with systematic search” to guarantee the comprehensiveness and reproducibility of the evidence (Snyder, 2019). Systematic searches were performed in PubMed/MEDLINE, Embase, Web of Science Core Collection, Cochrane CENTRAL, PsycINFO, and Scopus, covering the period from the inception of each database until September 2025, with a language restriction to English. The search approach included subject phrases and keywords, concentrating on three primary modules interconnected by Boolean logic: (1) Exercise dimension: “exercise,” “physical activity,” “aerobic,” “resistance,” “HIIT,” “endurance”; (2) Microbiota and metabolic dimension: “gut microbiota/microbiome,” “short-chain fatty acids/SCFAs,” “acetate/propionate/butyrate,” “FFAR2/FFAR3/GPR109A,” “microbiota-gut-brain axis”; (3) Psychological and cognitive outcomes: “depression,” “anxiety,” “mood,” “cognition,” “memory,” “executive function,” “stress reactivity.” Inclusion criteria: original research conducted on animals or humans; incorporating exercise-associated exposure or intervention; documenting at least one measurement of gut microbiota and/or Short-Chain Fatty Acid (SCFA); and reporting results related to emotional, cognitive, or stress-related factors. Concurrently, if the exposure/outcome criteria are satisfied, mechanistic investigations (including antibiotic de-germing, SCFA supplementation, receptor genetics, and microbiota transplantation) are prioritized. Exclusion criteria: only *in vitro* studies; interventions limited to probiotics/antibiotics without exercise components; absence of microbiota or SCFA data; or redundant publications lacking methodological rigor. To mitigate bias, reference tracing and citation tracking were employed, and independent screening and data extraction were performed by two researchers, with any discrepancies adjudicated by a third researcher. The search and screening procedure adhered to the PRISMA guidelines for documentation (Page et al., 2021; Rethlefsen et al., 2021). The quality of research and risk of bias were evaluated utilizing the Cochrane RoB 2 for randomized trials (Sterne et al., 2019), ROBINS-I for non-randomized studies (Sterne et al., 2016), and SYRCLE for animal experiments (Hooijmans et al., 2014). The aforementioned procedure offers methodological assurances for later evidence integration, mechanism chain organization, and the formulation of research agendas.

## 2 SCFAs: production, distribution, and measurement

SCFAs are primarily generated by the fermentation of fermentable dietary fibers in the colon by bacteria (van der Hee and Wells, 2021). The substrates comprise resistant starch, inulin/oligofructose, galactooligosaccharides, pectin, arabinan, and β-glucan, among others (Valdes et al., 2018). The substrate type and community ecology collectively influence the SCFA profile and yield: resistant starch and non-starch polysaccharides from whole grains are more conducive to butyrate synthesis, whereas inulin and pectin often elevate the levels of acetate and propionate (Lin et al., 2018). Key genera involved in butyrate production include *Faecalibacterium prausnitzii*, *Roseburia* spp., *Eubacterium rectale*, *Anaerobutyricum*/*Eubacterium hallii*, and *Subdoligranulum* (Louis and Flint, 2017). Propionate is synthesized by *Bacteroides*, *Veillonella*, *Phascolarctobacterium*, and *Prevotella*, among others (Reichardt et al., 2014). Acetate, the most prevalent SCFA, is produced by nearly all major anaerobic bacteria, with Bifidobacterium demonstrating notable efficiency via the “bifid shunt,” while *Akkermansia muciniphila* also generates acetate during mucin degradation (Belenguer et al., 2006; Cani et al., 2022). Interactions at cross-nutritional levels establish a stable fermentation network: lactic acid and acetate can be further transformed into butyrate by butyrate-producing bacteria, while succinate can be converted into propionate by specialized bacteria; concurrently, methanogens and sulfate-reducing bacteria enhance the thermodynamics of fermentation by eliminating hydrogen (Flint et al., 2015). Host factors, including an individual’s food composition, intestinal transit duration, bile acid reservoir, and mucus layer condition, can profoundly influence the efficacy and composition of SCFA synthesis (Bretto et al., 2025).

SCFAs establish a notable concentration gradient inside the intestinal lumen, with total concentrations in the proximal colon typically varying from 50 to 150 mmoL/L (van der Hee and Wells, 2021; Boets et al., 2017). The colonic epithelium absorbs SCFAs through monocarboxylate transporters MCT1 and MCT4, as well as sodium-coupled transporters SMCT1 and SMCT2 (Dirks et al., 2025). Butyrate serves as the principal energy substrate for colonic cells, supplying around 60–70% of their energy requirements. The oxidation diminishes the local oxygen partial pressure in the mucosa, fostering anaerobic ecology and reinforcing the barrier (Nireeksha et al., 2025). Absorbed SCFAs enter the liver through the portal vein and undergo substantial first-pass metabolism: propionate is primarily converted to glucose via gluconeogenesis, acetate is involved in lipid and cholesterol synthesis and is distributed to peripheral tissues and the brain, whereas butyrate is predominantly utilized by the mucosa and liver (Zheng et al., 2020; Perry et al., 2016). Consequently, the quantities in peripheral blood are significantly lower than those in the intestinal lumen: acetate ranges from around 50 to 200 μmoL/L, propionate from about 1 to 20 μmoL/L, and butyrate often measures < 1–5 μmoL/L (Boets et al., 2017). SCFAs traverse the blood-brain barrier through monocarboxylate transporters and influence neurons and glial cells at low micromolar concentrations, initiating receptor signaling and epigenetic modifications (Dirks et al., 2025); they furthermore modulate metabolism and inflammation in adipose tissue, skeletal muscle, and immune cells (Yang-Jensen et al., 2025). Fecal SCFAs represent the “unabsorbed remainder and lumen pool size” rather than the “production rate”; conversely, blood SCFAs indicate the dynamic equilibrium of “net appearance = production—tissue uptake/clearance—hepatic metabolism” (Byndloss et al., 2017). For estimating fluxes and tissue fates, stable isotope tracking, ^13^CO_2_ breath testing, and differential sampling of the portal and hepatic veins provide further insight (Xiao et al., 2024).

Matrix selection dictates the biological relevance and methodological approach in measurement. Feces represent the ecology of the intestinal lumen and upstream fermentation, significantly affected by diet, transit time, and moisture content (Amos et al., 2020); plasma/serum is more indicative of systemic availability and distal effects, yet exhibits low concentrations and is substantially influenced by first-pass metabolism and recent dietary or exercise conditions (Blaak et al., 2020). Gas chromatography-mass spectrometry (GC-MS) or gas chromatography-flame ionization detection is the conventional benchmark for detecting volatile fatty acids; liquid chromatography-tandem mass spectrometry (LC-MS/MS) with derivatization markedly improves sensitivity and selectivity for low-concentration blood and brain tissue samples (Wu et al., 2021; Trivedi et al., 2022). Utilizing stable isotope internal standards for matrix-matched calibration is advisable to rectify recovery rates and matrix effects (Mosley et al., 2024). Pre-analytical processing is essential: post-collection, immediate cessation of fermentation and chemical reactions must be ensured through cooling, acidification, or the application of metabolic inhibitors within minutes, followed by storage at –80°C and uninterrupted cold chain transport (Mirzayi et al., 2021); fecal samples should be meticulously homogenized and standardized by dry weight, with documentation of Bristol stool type and sampling-intake-exercise intervals (Hold et al., 2014); for blood samples, plasma is preferred, and fasting/postprandial conditions along with the duration from blood collection to centrifugation should be standardized, considering the influence of anticoagulants on detection (Wang H. et al., 2025). Quality control must encompass method blanks, matrix-spiked recoveries, duplicate samples, within-batch and between-batch quality controls, and should give the linear range, detection limit, and quantification limit. Cross-study comparability relies on the documentation of essential metadata, including derivatization reagents, column types, internal standards, calibration models, sample moisture content, and daily defecation volume (Deng et al., 2021; Table 1).

**TABLE 1:** Methodological and quality control recommendations for SCFA quantification.

Dimension	Recommendations	Key parameters	Potential issues
Internal standard (IS) selection	Prioritize stable isotope-labeled IS with identical label positions and numbers (Sun et al., 2020).	Co-elution with analyte (ΔRT < 0.05 min); high isotopic purity.	Issue: ^2^H-IS chromatographic shifts; MRM overlap. Action: List IS source, purity, and analyte-IS pairs.
IS Spiking Time	Spike IS pre-extraction and pre-derivatization (Xing et al., 2025).	IS concentration comparable to endogenous analyte levels.	Issue: Post-extraction spiking fails to correct process losses. Action: Report IS concentration, volume, and exact spiking point.
Calibration strategy	Use matrix-matched external calibration with IS correction, or standard addition method for severe matrix effects (Trivedi et al., 2022).	≥ 6 Points; *r*^2^ ≥ 0.995; 1/x or 1/*x*^2^ weighting.	Issue: Solvent-only curves cause underestimation due to matrix suppression. Action: Specify model, weighting, and range.
Linearity and dynamic range	Establish linearity using analyte/IS response ratio. Use segmented or quadratic regression if needed (Mauthe et al., 2025).	Back-calculated accuracy: ± 15% ( ± 20% at LLOQ).	Issue: Non-linearity at range extremes. Action: Report linear range, weighting, and back-calculated errors.
LOD/LOQ determination	Determine via S/N ratio or statistical methods on low-concentration samples (Pum, 2019).	S/N ≥ 3 for LOD, ≥ 10 for LOQ; CV ≤ 20% at LLOQ.	Issue: Instrument noise alone is insufficient; matrix noise must be included. Action: Specify method, matrix, and final validated values.
Matrix effect (ME) assessment	Evaluate via post-extraction spike or Matuszewski’s method (Arnold and Booth, 2022).	ME: 80–120%; CV ≤ 15%.	Issue: Inter-individual/batch ME variability. Action: Report matrix source, n, mean ME (%), and CV (%).
Recovery / derivatization yield	Compare pre- vs. post-extraction spiked samples to assess overall process efficiency (Trivedi et al., 2022).	Consistent recovery (CV ≤ 15%) is critical.	Issue: High but inconsistent recovery is unreliable. Action: Report mean recovery ± SD and inter-batch CV.
Isotopic interference	Evaluate and correct for natural isotopic abundance crosstalk in MRM transitions if necessary (Huang et al., 2024).	Select interference-free transitions or apply a correction matrix.	Issue: Derivatization may introduce unpredicted mass shifts. Action: List MRM pairs, CEs, and state if correction was applied.
Drift and inter-batch consistency	Intersperse matrix QC samples (low, mid, high) throughout analytical runs to monitor system stability (Wang J. et al., 2025).	QC values within ± 2 SD of established mean.	Issue: Signal drift in long batches. Action: Report QC pass rate and any drift correction methods used.
Dilution integrity	Validate sample dilution procedures for concentrations above the ULOQ (Mauthe et al., 2025).	Accuracy within ± 15% after dilution.	Issue: Mismatched dilution solvent can introduce bias. Action: Report dilution factors, diluent, and acceptance criteria.
Cross-matrix comparability	Validate and establish separate matrix-matched curves for each distinct matrix (e.g., feces, plasma) (Bar et al., 2020).	Do not extrapolate calibration from one matrix to another.	Issue: Using a single curve for different matrices causes systematic error. Action: Provide separate validation data for each matrix.
Traceability and reference materials	Use certified reference materials (CRMs) if available, or establish stable, well-characterized in-house QCs (Lippa et al., 2022).	Use large, homogenized matrix pools as long-term QCs.	Issue: In-house QCs may lack universal transferability. Action: Document QC preparation, characterization, and stability.
Data processing and interpretation	Standardize and pre-define rules for blank subtraction, outlier tests, and re-analysis criteria (Mulder et al., 2023).	Define acceptance limits for IS response, replicate agreement, etc.	Issue: Lack of clear rules compromises data integrity. Action: Report data rejection criteria and processing workflow.
Critical metadata logging	Document all critical experimental details (reagent lots, instrument conditions, storage parameters) (Mirzayi et al., 2021).	Ensure full reproducibility.	Issue: Missing metadata prevents replication. Action: Provide a comprehensive metadata list, ideally as Supplementary material.

We must forcefully emphasize the critical distinction between fecal SCFA concentration which merely represents a static snapshot of unabsorbed luminal residue and the actual production rate or systemic flux. The field’s current over-reliance on static fecal SCFAs as a proxy for systemic physiological effects is a major source of inconsistency and a profound barrier to progress. Therefore, as a call to action, future research must prioritize dynamic measures to truly understand SCFA kinetics and systemic exposure. To achieve this, we strongly advocate for the integration of several advanced methodologies. For instance, stable isotope tracer studies utilizing approaches such as 13C-labeled fermentable fibers can precisely track the dynamic fate of SCFAs from microbial ingestion and production to fecal excretion and their appearance in the systemic circulation. Additionally, portal vein sampling in applicable preclinical models provides the most accurate and direct measurement of the actual SCFA flux entering the host’s body prior to hepatic first-pass metabolism. Furthermore, human clinical trials must move beyond single fasting blood draws by implementing repeated postprandial and post-exercise sampling to accurately capture the dynamic fluctuations in circulating SCFAs. Additionally, standardizing and documenting the FITT components and fermentable fiber consumption is essential to enhance the stability, reproducibility, and translational significance of the findings (Mailing et al., 2019).

## 3 Mechanistic pathways linking SCFAs to brain function

### 3.1 Receptor-mediated signaling: FFARs and beyond

SCFAs facilitate gut-brain communication via a set of G protein-coupled receptors (GPCRs), primarily involving FFAR2 (GPR43), FFAR3 (GPR41), and GPR109A (HCAR2), and may additionally modulate “olfactory-like” receptors such as OR51E2/Olfr78, thereby establishing a broadly distributed and complementary signaling network (Dalile et al., 2019; Table 2). FFAR2 and FFAR3 are prominently expressed in enteroendocrine and enterochromaffin cells within the small intestine and colon, as well as in non-endocrine intestinal epithelial cells, goblet cells (Parada Venegas et al., 2019b), and colonic cells. In the immune system, their expression is noted in neutrophils, monocytes/macrophages, dendritic cells, and certain T cell subsets, where they play a role in regulating inflammation and tolerance (Hu et al., 2022). In the peripheral nervous system, FFAR3 is found in enteric nervous system neurons, vagal nodal neurons, and sympathetic ganglia, while FFAR2 has also been identified in some sensory neurons and glial cells (Martin et al., 2018). GPR109A is predominantly localized in colonic epithelium and mucosal immune cells (Zhu et al., 2023), exhibiting functional expression in enteroendocrine L cells and peripheral immune cells. In terms of ligand preference, acetic acid predominantly activates FFAR2, propionic acid exhibits a greater affinity for FFAR3, and butyric acid can engage both FFAR2 and FFAR3 while also activating GPR109A with enhanced efficacy (Mukhopadhya and Louis, 2025); furthermore, propionic acid and acetate may activate OR51E2/Olfr78, potentially influencing the regulation of vascular and smooth muscle tone (Pluznick, 2014). Regarding receptor coupling, FFAR2 can associate with both Gi/o and Gq/11 pathways, FFAR3 mostly inhibits adenylate cyclase via Gi/o, while GPR109A predominantly couples with Gi/o (Dalile et al., 2019; He et al., 2018). The aforementioned tripartite distinctions in “receptor-coupling-ligand” establish a molecular foundation for SCFAs to elicit distinct hormonal, neural, and immunological responses across various cell types; additionally, different receptors may demonstrate biased agonism and heterodimerization, hence enhancing signal variety (Zhang et al., 2024).

**TABLE 2:** Overview of the SCFA-mediated GPCR network.

Receptor	Primary ligands	Coupling	Representative expression	Key functions	Remarks
FFAR2	Acetate > propionate (Rooks and Garrett, 2016)	Dual Gi/o and Gq/11 (↓cAMP, ↑Ca^2+^) (Bolognini et al., 2016)	Intestine: EECs, colonocytes, goblet cells (Brooks et al., 2017). Immune: Neutrophils, monocytes, macrophages, DCs (Wang et al., 2024). Nervous: Sensory/glial cells (Dalile et al., 2019).	Integrates endocrine and immune signals; modulates inflammation and tolerance; gut-nerve sensory axis modulation (Jiang et al., 2025).	Ligand preference is context-dependent; functionally complementary to FFAR3 (Xu et al., 2019).
FFAR3	Propionate > Butyrate (Rooks and Garrett, 2016)	Primarily Gi/o (↓cAMP) (Bolognini et al., 2016)	Intestine: EECs (Brooks et al., 2017). Nervous: Enteric neurons, vagal nodose and sympathetic ganglia (Dalile et al., 2019).	Gut-brain axis signaling; regulates enteric neural circuits; endocrine and metabolic control (Dalile et al., 2019; Silva et al., 2020).	Co-expression with FFAR2 allows for differential cellular outputs (Xu et al., 2019).
GPR109A	Butyrate (Zhu et al., 2023)	Primarily Gi/o (Mukhopadhya and Louis, 2025)	Intestine: Colonic epithelium, EECs (Zhu et al., 2023). Immune: Mucosal and peripheral immune cells (Coutzac et al., 2020).	Promotes epithelial/mucosal homeostasis; regulates gut barrier function and tolerance (Zhu et al., 2023; Coutzac et al., 2020).	Actions prominent in epithelial and immune compartments; tightly linked to SCFA metabolism (Mukhopadhya and Louis, 2025).
OR51E2/Olfr78	Propionate / acetate (Pluznick, 2014)	GPCR (Pluznick, 2014)	Vasculature and smooth muscle (Yang et al., 2023).	Potential regulation of vascular tone (Yang et al., 2023).	Evidence is primarily from animal models; serves a complementary systemic role (Pluznick, 2014).

SCFAs stimulate the secretion of PYY and GLP-1 from L cells via FFAR2/FFAR3/GPR109A at the level of enteroendocrine cells (Dalile et al., 2019). The Gq/11 pathway of FFAR2 elevates intracellular Ca2+ levels and initiates vesicle exocytosis; the Gi/o pathway of FFAR3 diminishes cAMP and collaborates with voltage-gated Ca2+ channels to enhance secretion; the Gi/o signaling of GPR109A is associated with the cell’s metabolic state, modulating the transcription and release of glucagon-like peptides (Christiansen et al., 2018; Psichas et al., 2015). Distinct SCFAs exhibit varying secretion profiles: propionate typically exerts a more pronounced influence on PYY/GLP-1, while acetate proves effective at elevated local concentrations (Larraufie et al., 2018); butyrate, beyond receptor engagement, may also enhance precursor gene expression via epigenetic mechanisms (refer to 3.2), demonstrating a dual role of “immediate secretion + transcriptional remodeling” (Garcia-Serrano et al., 2024). PYY interacts with vagal afferent terminals and brainstem integration regions via Y2 receptors, simultaneously decelerating gastric emptying and diminishing appetite; GLP-1 not only stimulates insulin secretion but also enhances glucose homeostasis, suppresses feeding, and modulates lipid metabolism through the vagal afferent and central GLP-1 receptor pathways (Yan et al., 2023). Recent findings regarding “neuropods” indicate that endocrine cells can establish synapse-like connections with vagal afferents, and the stimulation of FFAR receptors can alter the discharge of vagal fibers within milliseconds to seconds, facilitating rapid transmission from the intestinal lumen to the nucleus tractus solitarius (NTS) (Kaelberer et al., 2018); intestinal peptides function as both paracrine mediators and signal amplifiers in this mechanism, collaborating with direct neural modulation to influence energy and behavioral phenotypes (Ohara and Hsiao, 2025).

SCFAs can serve as immediate stimulants for enterochromaffin cells and 5-hydroxytryptamine (5-HT), while also modulating the production of enzymes like tryptophan hydroxylase 1 (TPH1), so demonstrating a combined action of receptor-mediated and epigenetic control (Stasi et al., 2019). The activation of FFAR2/FFAR3 induces fast Ca2+ influx and 5-HT vesicle exocytosis; butyric acid can also augment TPH1 transcription via GPR109A and HDAC inhibition, hence improving 5-HT availability (Liu et al., 2022). The released 5-HT in the mucosa directly stimulates vagal afferent terminals via 5-HT3 receptors, swiftly influencing gastrointestinal motility, satiety, and stress responses (Bellono et al., 2017); conversely, it enhances intestinal peristalsis and secretion through 5-HT4 and other receptors, indirectly impacting nutrient absorption and glucose metabolism (Mawe and Hoffman, 2013). In summary, SCFAs establish a coupling chain of “receptor-second messenger-neurotransmitter/hormone release-vagal afferent” on endocrine cells and sensory neurons via FFAR2, FFAR3, and GPR109A, consequently affecting hunger, energy balance, glucose homeostasis, and autonomic nerve activity (He et al., 2018). This process is meticulously governed by factors including substrate type, luminal concentration gradient, receptor expression profile, and timing of feeding/exercise, indicating a viable strategy of “targeting FFARs—optimizing intestinal peptide and 5-HT signaling—enhancing vagal afferent” in the intervention of exercise and nutrition combination (Wang H. et al., 2025).

### 3.2 Epigenetic modulation in the brain

SCFAs traverse the blood-brain barrier via monocarboxylate transporters, facilitating a connection between metabolism and epigenetics in neurons, astrocytes, and microglia (Silva et al., 2020). Butyric acid serves as a prominent inhibitor of class I and IIa histone deacetylases (HDACs), and at physiological-pharmacological concentrations, it can augment the acetylation of various sites on H3/H4, enhance chromatin accessibility, and elevate promoter/enhancer activity (Hays et al., 2024); propionic acid exhibits a weaker yet analogous HDAC inhibitory effect (Silva et al., 2020); acetic acid primarily facilitates acetylation reactions by providing acetyl-CoA (Ac-CoA) as a donor, with nuclear-localized acetyl-CoA synthetase 2 (ACSS2) in neurons being particularly vital in this mechanism (Li et al., 2017). These modifications are incorporated into the CREB-p300/CBP pathway, enhancing the transcription of neurotrophic and plasticity-associated genes (Kennedy et al., 2017). Butyric acid can also integrate into the TCA cycle, supplying energy and substrate for the Ac-CoA pool and acetylation processes, exemplifying a dual mechanism of “substrate availability + enzyme inhibition” (Mukhopadhya and Louis, 2025).

**FIGURE 2:**
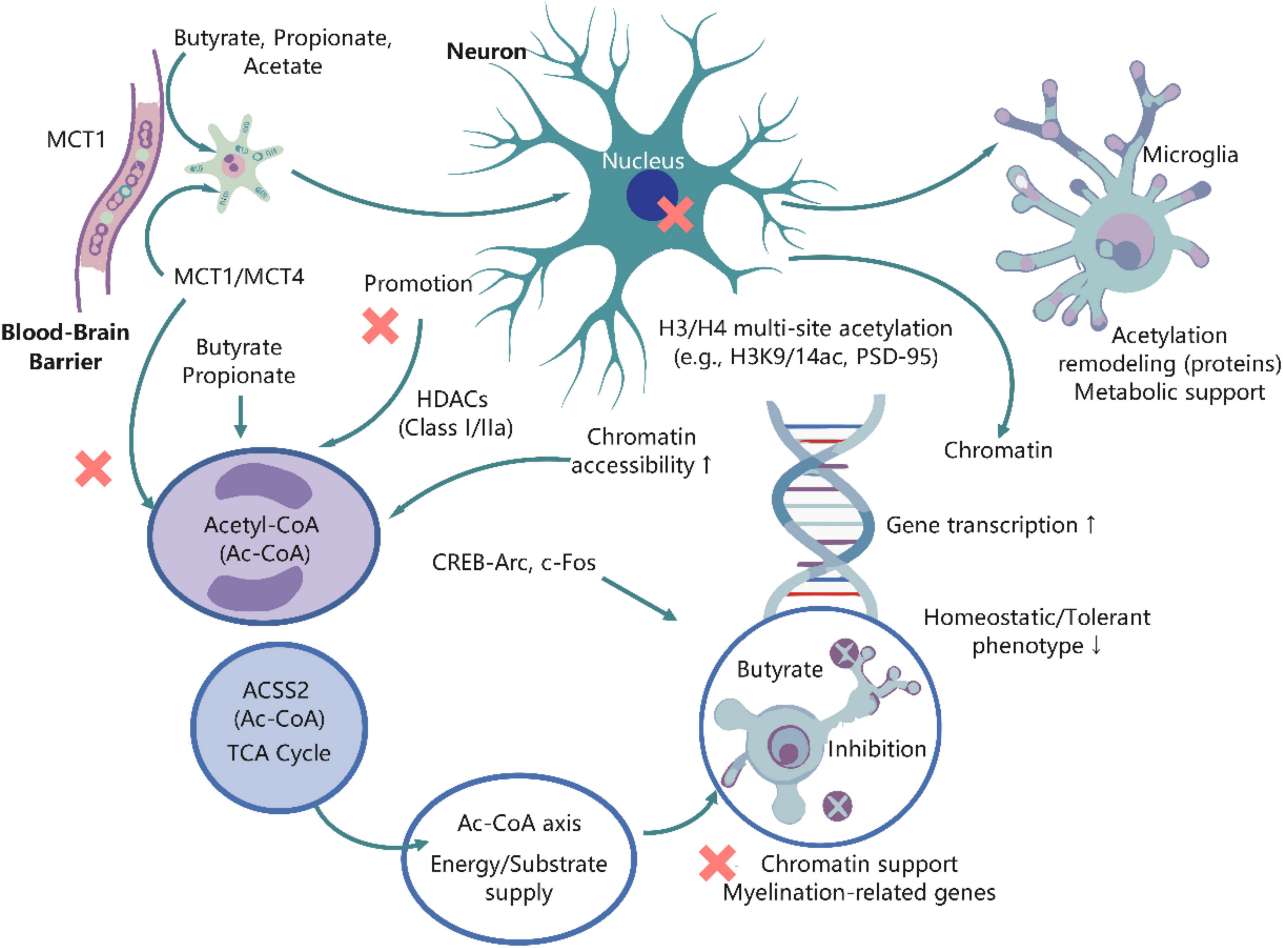
SCFAs regulate brain function within the central nervous system through “metabolic-epigenetic coupling.” After crossing the blood-brain barrier via monocarboxylate transporters, SCFAs regulate gene expression in different neural cell types. In neurons, they act as endogenous histone deacetylase inhibitors, promoting an open chromatin state that upregulates neuroplasticity genes like BDNF. Concurrently, SCFAs provide essential metabolic substrates for mitochondrial respiration. In glial cells, they modulate microglia toward an anti-inflammatory homeostatic phenotype and support astrocyte metabolic coupling, collectively promoting neurogenesis, enhancing synaptic plasticity, and mitigating neuroinflammation. Figure was created with EdrawMax.

At the functional level, the heightened acetylation propensity resulting from HDAC inhibition typically upregulates neurotrophic and synaptic plasticity genes, facilitating dendritic spine formation, the consolidation of long-term potentiation (LTP), and adult hippocampal neurogenesis, and correlates with enhanced emotional and cognitive stability (Ohara and Hsiao, 2025). In astrocytes and oligodendrocyte lineages, acetylation remodeling facilitates the regulation of genes associated with metabolic support and myelination (Xu et al., 2024); in microglia, butyric acid directs chromatin toward a “steady-state/tolerant” phenotype via HDAC inhibition, diminishing adverse marks at the promoters of pro-inflammatory genes and curtailing excessive activation (supplementing the immune regulation in Section 3.3) (Matt et al., 2018). In addition to acetylation, SCFAs, via their respective acyl-CoA intermediates, can initiate other novel histone acylations, such as butyrylation (Kbu), propionylation (Kpr), and crotonylation (Kcr) (Fellows et al., 2018). These modifications are dynamically synthesized by “writers” like p300/CBP under varying acyl-CoA donor conditions and selectively identified by “readers” such as bromodomain and YEATS domain proteins, frequently resulting in robust transcriptional activation or enrichment of immediate early genes and activity enhancers (Sabari et al., 2017). Due to variations in metabolic flux and receptor/enzyme expression across distinct brain regions and cell types, the acylation profiles influenced by SCFAs are specific to both region and cell type, offering novel targets and assessment parameters for precision nutrition and integrated exercise-nutrition interventions (Yan et al., 2023). SCFAs enhance epigenetic health and neuroplasticity in the brain via a triad of mechanisms: “HDAC inhibition, Ac-CoA substrate provision, and varied acylation marks,” hence fostering beneficial cognitive and emotional functioning (Bourassa et al., 2016; Figure 2).

### 3.3 Immune and barrier regulation

Regarding barrier function, SCFAs serve as the primary energy substrate for colonic epithelium. Their oxidation diminishes local oxygen partial pressure, stabilizes HIF-1α signaling, and enhances the production of barrier-protective genes (Wang R. X. et al., 2021). Butyrate facilitates the formation and preservation of tight junction complexes by activating AMPK and modulating PPARγ, enhances the expression of Occludin, Claudin-1, and ZO-1, and inhibits MLCK-mediated actin contraction, thereby diminishing epithelial permeability and averting “leaky gut” (Silva et al., 2020; Jourova et al., 2022). Concurrently, SCFAs facilitate the synthesis and secretion of mucin (MUC2) in the epithelium and goblet cells via FFAR2/FFAR3 and GPR109A, augment the local efficacy of antimicrobial peptides and secretory IgA, and reinforce both physical and chemical barriers (Bolognini et al., 2016; Chen et al., 2020). GPR109A signaling additionally facilitates the production of epithelial-derived repair factors, including IL-18, hence augmenting wound healing and barrier regeneration capabilities (Zhang et al., 2022).

SCFAs influence immunity by establishing a tolerant yet effective immunological state via a triad of mechanisms: metabolic reprogramming, receptor signaling, and epigenetics (Lavelle and Sokol, 2020). Initially, SCFAs stimulate FFAR2/FFAR3 and GPR109A, prompting dendritic cells to adopt a tolerant phenotype and facilitating the differentiation and proliferation of peripheral inducible Tregs; butyric acid and propionic acid augment the accessibility and stable expression of the FOXP3 locus via HDAC inhibition (Luu et al., 2019; Luu et al., 2021). Secondly, in macrophages and neutrophils, SCFAs impede NF-κB activation and the excessive release of pro-inflammatory mediators (TNF, IL-6, IL-1β), while enhancing mitochondrial oxidative metabolism and antioxidant capacity via AMPK, so restraining the aberrant activation of NLRP3 inflammasomes (Zhou et al., 2017). Thirdly, SCFAs facilitate B cell development and IgA synthesis, enhancing the mucosal immunological barrier and maintaining microbial community equilibrium (Hayashi et al., 2021). These actions do not constitute a “comprehensive suppression of immunity”; instead, they mitigate superfluous inflammation while preserving robust protection against pathogens, so attaining a dynamic equilibrium within the immune system (Lavelle and Sokol, 2020).

The beneficial alterations in the intestinal mucosa and systemic immunity may influence the neurological system, creating a triadic “gut-immune-brain” protective effect (Morais et al., 2021). An enhanced intestinal barrier diminishes the systemic leakage of bacterial byproducts and inflammatory mediators, mitigating the effects of peripheral inflammation on the blood-brain barrier (Parker et al., 2020). SCFAs and their subsequent anti-inflammatory signals can fortify the tight junctions of brain microvascular endothelial cells (including Occludin and Claudin-5) and sustain a “homeostasis/repair” expression profile in microglia via dual mechanisms of receptor and HDAC inhibition, thereby curtailing excessive activation and neuroinflammation (Fusco et al., 2025); Chen et al., 2025). Consequently, a low-inflammatory and highly robust neuronal internal environment is established, facilitating sustained synapse function, cognition, and emotion (Shin et al., 2025).

### 3.4 Vagus nerve communication

The vagus nerve functions as a fast conduit in the gut-brain axis, swiftly relaying the metabolic condition of the intestinal lumen to the brainstem and higher neural regions (Berthoud, 2008). SCFAs influence vagal afferent signals by two distinct mechanisms: one involves direct action on peripheral sensory neurons, while the other entails indirect modulation through intestinal peptides and serotonin (5-HT) generated by enteroendocrine and chromaffin cells (Loh et al., 2024). The two systems exhibit unique properties regarding time scale, receptor spectrum, and signal encoding strategies (Kaelberer et al., 2018).

Vagal nodal neurons exhibit functioning FFAR3 (GPR41) and other receptors in the direct pathway (Cao et al., 2024). SCFAs can stimulate specific subgroups of vagal afferents, modifying their discharge frequency and responsiveness to mechanical and chemical stimuli (Wallrapp and Chiu, 2024). This mechanism mostly occurs via the Gi/o pathway, which regulates ion channels and second messengers, therefore affecting the rapid response of afferent terminals to alterations in the intestinal wall environment (Jacobson et al., 2021). Furthermore, local pH fluctuations and metabolic coupling may subtly affect neuronal excitability via non-GPCR systems, including ASICs and TRPV, so offering supplementary physiological control points for the SCFA-vagus interaction (Wachsmuth et al., 2022).

The indirect pathway focuses on enteroendocrine and enterochromaffin cells. SCFAs activate FFAR2, FFAR3, and GPR109A on L cells, prompting the secretion of PYY and GLP-1 (Yadav et al., 2013). These intestinal peptides interact with vagal afferent terminals and nodal neurons via Y2 receptors and GLP-1 receptors, respectively, producing robust signals for satiety and metabolic equilibrium (Trapp and Brierley, 2022). Concurrently, SCFAs facilitate the production and secretion of 5-HT in enterochromaffin cells (Spencer and Keating, 2025). The release of 5-HT on the mucosal side directly stimulates the activation of vagal afferents via 5-HT3 receptors, offering immediate feedback on gastrointestinal motility and fullness (Hwang and Oh, 2025). Endocrine cells possessing “neuropods” can establish synapse-like connections with vagal afferents, facilitating the rapid transmission of chemical information within milliseconds to seconds using fast neurotransmitters like glutamate and ATP (Kaelberer et al., 2018). Subsequently, peptides such as PYY and GLP-1 offer prolonged enhancement and integration within minutes. The amalgamation of these two temporal intervals renders the signal both versatile and resilient (Kaelberer et al., 2018; Singh, 2025).

At the central integration level, vagal afferents initially undergo primary processing in the NTS of the medulla oblongata, subsequently converging into the regulatory network through various pathways: through the parabrachial nucleus and the hypothalamus to affect appetite and energy balance; through the locus coeruleus to modulate arousal, attention, and stress response; and in conjunction with the amygdala and the prefrontal cortex to engage in emotion evaluation, reward regulation, and executive function (Berthoud, 2008; Luo et al., 2023). Simultaneously, the reflex arc established by the NTS and the dorsal motor nucleus of the vagus can inhibit gastrointestinal motility, pancreatic secretion, and hepatic glucose metabolism, so creating a closed-loop regulation of “gut-brain-gut” (Clyburn and Browning, 2021). The cholinergic anti-inflammatory route of the vagus nerve can limit excessive peripheral inflammation, thereby complementing the barrier and immunological optimization outlined in section 3.3, and collectively fostering a low-inflammatory and homeostatic interior milieu (Han et al., 2017).

**FIGURE 3:**
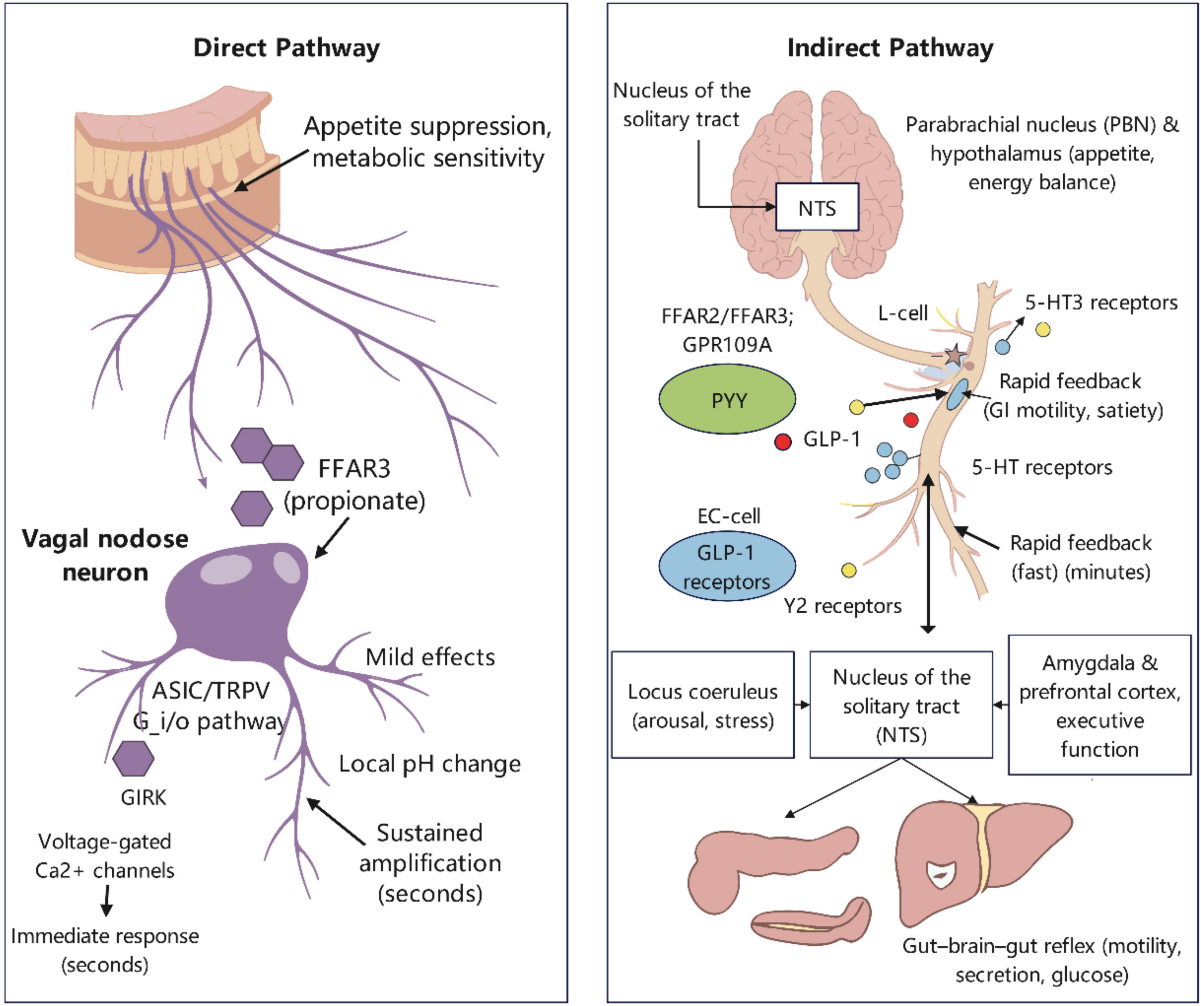
SCFAs regulate vagal afferent signals via direct and indirect pathways. SCFAs produced in the gut lumen influence vagal signaling through two complementary routes. Direct Pathway: SCFAs (primarily propionate) cross the epithelial barrier and directly activate FFAR3 receptors expressed on vagal afferent nerve terminals in the lamina propria. Indirect Pathway: SCFAs activate FFAR2/3 and GPR109A on enteroendocrine cells (EECs), stimulating the release of gut peptides (GLP-1, PYY) and on enterochromaffin cells, stimulating the release of serotonin (5-HT). These signaling molecules then activate their respective receptors (GLP-1R, Y2R, 5-HT3R) on vagal afferents. These signals are integrated in the nucleus tractus solitarius (NTS), influencing downstream brain regions involved in mood, cognition, and stress regulation, while also forming a gut-brain-gut feedback loop via the dorsal motor nucleus of the vagus (DMV). Figure was created with EdrawMax.

In summary, SCFAs distinctly influence the intensity, timing, and coding patterns of vagal afferent input via a dual mechanism of “direct neural modulation and gut peptide/serotonin mediation” (Loh et al., 2024; Figure 3). The NTS functions as a central hub, converting visceral impulses into coordinated responses across various systems, resulting in beneficial results such as the suppression of overeating, the optimization of glucose and lipid metabolism, and the improvement of stress adaption and emotional stability (Clyburn and Browning, 2021; Cryan et al., 2019). This process is meticulously governed by factors including the type and concentration gradient of SCFAs, receptor expression profiles, intestinal barrier integrity, and microbial composition. This indicates that lifestyle modifications involving dietary fiber, consistent routines, and moderate exercise can enhance physical and mental wellbeing, as well as social cohesion, via the “gut-vagus-brain” pathway (Loh et al., 2024).

## 4 The exercise-microbiome-SCFA axis: evidence from animal studies

### 4.1 Exercise reshapes the microbiota, favoring SCFA functions

Consistent moderate-intensity aerobic training in rodent models remodels the gut microbiota toward a short-chain fatty acid-producing phenotype (Qi et al., 2022). This is characterized by elevated alpha diversity, improved community stability, and a strengthened anaerobic niche due to luminal acidification and diminished redox potential (Wang B. et al., 2021). This remodeling promotes the proliferation of essential SCFA-producing taxa, encompassing groups such as Lachnospiraceae and Ruminococcaceae, along with significant genera including Butyricicoccus, Roseburia, Anaerostipes, and Blautia (Min et al., 2024; Lin et al., 2025). Simultaneously, cross-feeding species such as Akkermansia and Bifidobacterium are also enhanced (Carey and Montag, 2021). Metagenomic and transcriptomic analyses indicate an upregulation of carbohydrate-active enzymes (CAZymes) involved in fiber degradation and an enrichment of pathways for butyrate and propionate synthesis, notably increasing the transcriptional activity of butyryl-CoA: acetate CoA-transferase and butyrate kinase genes (Yan et al., 2024).

The physiological advantages of this exercise-induced SCFA increase are diverse. Butyrate, a principal energy source for colonocytes, enhances intestinal barrier integrity by augmenting epithelial mitochondrial respiration, hence stabilizing mucosal hypoxia and promoting the upregulation of tight junction proteins and mucin synthesis through HIF-1α, HDAC inhibition, and GPR109A signaling pathways (Iliev et al., 2025; Liu et al., 2018). Propionate, absorbed in the portal vein, facilitates hepatic gluconeogenesis and enhances satiety by stimulating the production of PYY and GLP-1 (Décarie-Spain et al., 2024). Acetate systematically contributes to immunological and energy regulation (Mukhopadhya and Louis, 2025). The microbial changes are accompanied by host physiological adaptations, such as enhanced mucosal perfusion and modified intestinal transit times, which maximize substrate availability for fermentation in the proximal colon, hence supporting a beneficial SCFA profile (Clauss et al., 2021).

Increasing data substantiates the causal relationship among exercise, microbiota, and host health. Fecal microbiota transplantation from selected donors mimics the enrichment of SCFA-producing bacteria and enhances metabolic outcomes in recipients, but antibiotic or germ-free models negate the advantages of exercise (Qi et al., 2022; Allen et al., 2018). The dose-response exhibits a “U-shaped” curve, indicating that moderate, consistent training is optimal, whereas excessive high-intensity exercise may be harmful (Cintado et al., 2025). The extent of these effects is influenced by factors like age, diet, and host genetics (O’Sullivan et al., 2015). Moderate aerobic activity facilitates a multi-faceted “exercise-microbiota-SCFA-host” axis, fostering a symbiotic condition that improves intestinal barrier integrity and systemic metabolic equilibrium (Table 3).

**TABLE 3:** Multi-level mechanisms of exercise-induced modulation of gut SCFAs.

Regulatory level	Core mechanisms	Key molecules and pathways	Ecological and physiological consequences
Microbiota community and function	Niche remodeling: ↑α/β diversity, ↑ community stability, ↓ luminal pH (Wang B. et al., 2021). Taxa enrichment: ↑ Butyrate/propionate producers (Min et al., 2024; Lin et al., 2025). Functional upregulation: ↑ CAZyme repertoire; ↑ expression/activity of butyrate synthesis genes. Enhanced cross-feeding: ↑ Lactate→propionate/butyrate and acetate→butyrate pathways (Carey and Montag, 2021).	Microbiota: *Lachnospiraceae, Ruminococcaceae, Roseburia, Akkermansia*. Genes/Enzymes: CAZymes, *but*, *buk*. Metabolites: lactate, acetate.	Increased total SCFA yield and production rate. Optimized SCFA profile (↑ butyrate/propionate ratio). Enhanced community metabolic efficiency and resilience.
Host physiology and gut environment	Optimized GI motility: Modulated transit time increases substrate availability and fermentation window (Clauss et al., 2021). Mucosal adaptation: ↑ Mucosal perfusion and mucus turnover provides endogenous substrates (Mach and Fuster-Botella, 2017). Physicochemical modulation: stabilized anaerobic gradient inhibits putrefactive bacteria.	Processes: Intestinal transit, mucosal blood flow. Signals: Bile acids, gut peptides.	Improved substrate-microbe matching. Favorable environment for efficient SCFA production.
Gut barrier and SCFA absorption	Barrier reinforcement: ↑ Tight junction (TJ) proteins and MUC2 expression (Iliev et al., 2025). Upregulated transport: ↑ MCT1 and SMCT1 expression in colonocytes (Gyimesi et al., 2020). Epithelial metabolic coupling: Butyrate fuels colonocytes, stabilizing local hypoxia (Liu et al., 2018).	Proteins: Occludin, Claudins, MUC2. Transporters: MCT1, SMCT1. Pathways: HIF-1α, GPR109A, HDAC inhibition.	Increased net SCFA flux into portal circulation. Reinforced gut barrier integrity. Positive “production-absorption-barrier” synergy.
Systemic circulation and tissue utilization	Modulated hepatic metabolism: Altered SCFA proportions entering systemic circulation (Décarie-Spain et al., 2024). Enhanced peripheral utilization: ↑ circulating SCFAs; ↑ uptake and oxidation in skeletal muscle (Mukhopadhya and Louis, 2025). Amplified systemic signaling: strengthened receptor-mediated effects in immune, metabolic, and neural tissues (Dalile et al., 2019).	Metabolism: Gluconeogenesis. Transport: MCT1, MCT4. Receptors: FFAR2, FFAR3. Hormones: GLP-1, PYY.	Improved SCFA bioavailability for distant target organs. Provides material basis for systemic regulatory roles.
Moderating factors and conditions	Dose-response: Optimal effects with moderate-intensity aerobic training (Cintado et al., 2025). Individual variability: Effects modulated by age, sex, diet, and baseline microbiota (O’Sullivan et al., 2015). Methodological rigor: Results depend on sampling, analytics, and covariate control.	Training: Intensity, duration, frequency. Host: Age, sex, diet. Study design: standardized protocols, multi-omics integration.	Emphasizes need for precision exercise interventions. Provides framework for study design and data interpretation.

### 4.2 Exercise enhances the supply and accessibility of SCFAs in the colon and circulation

Prolonged aerobic training reliably increases SCFA concentrations in the gastrointestinal lumen and bloodstream, but the extent varies (Lin et al., 2025). This action arises from a combinatorial augmentation of the dynamics of SCFA synthesis, absorption, and barrier function (Tomkovich and Jobin, 2016). Exercise modifies the microbiota to enhance mutualistic cross-feeding pathways (e.g., lactate/acetate to butyrate) and increases the expression of essential fermentation enzymes (Yan et al., 2024). Simultaneously, enhanced gastrointestinal motility and augmented mucus secretion elevate substrate availability in the proximal colon (Qin et al., 2022), while a slight decrease in luminal pH inhibits putrefaction, thereby maintaining the SCFA reservoir (Sanz et al., 2025). This establishes a reliable and consistent supply of acetate, propionate, and butyrate for host absorption.

In terms of absorption and barrier function, increased luminal SCFAs stimulate the expression of monocarboxylate transporters (MCT1, SMCT1) in the colonic epithelium, enhancing their uptake (Abdelhalim, 2024). Butyrate, as an optimal energy source for colonocytes, improves mitochondrial function, decreases local oxygen tension, and fortifies the intestinal barrier by upregulating tight junction proteins and mucin (Jourova et al., 2022; Litvak et al., 2018). This induces a condition characterized by low inflammation and low permeability. Despite the disruptive nature of acute high-intensity exercise, adaptive enhancements in mesenteric microcirculation during recovery phases ultimately enhance the efficiency of SCFA delivery into the portal circulation, thereby reinforcing the gut-liver axis (Mach and Fuster-Botella, 2017).

The systemic effects of SCFAs are further influenced by hepatic first-pass metabolism and adaptations in peripheral tissues. Propionate is primarily utilized for hepatic gluconeogenesis, while butyrate is metabolized by colonocytes; in contrast, acetate more efficiently reaches systemic circulation (Mukhopadhya and Louis, 2025). Endurance training improves the consumption of SCFAs in skeletal muscle and increases the sensitivity of peripheral tissues to their signals, hence enhancing metabolic and immunological advantages (Jollet et al., 2021). This complex “fermentation-absorption” coupling is additionally regulated by the bile acid-gut peptide axis (e.g., FXR/TGR5, GLP-1/PYY), creating a positive feedback loop (Sun et al., 2018). Nevertheless, evaluating these effects necessitates stringent methodological control, considering nutrition, sample period, and analytical procedures (Hughes and Holscher, 2021). Ultimately, progressive endurance training, together with sufficient nutrition and recovery, enhances the SCFA supply chain, establishing a robust biological basis for exercise-induced metabolic wellness (Scheiman et al., 2019).

### 4.3 Exercise-induced behavioral and neural plasticity benefits mediated by SCFAs

Multifaceted intervention studies demonstrate a causal relationship among exercise, SCFAs, and cognitive performance, notably in relation to stress and anxiety (Liu et al., 2019). Research utilizing microbiota-depletion mice indicates that the behavioral advantages of exercise are frequently diminished in the absence of a gut microbiome and can be partially reinstated through supplementation with SCFAs such as butyrate or propionate (Godinho et al., 2024). This restoration is accompanied by the reestablishment of molecular indicators of hippocampal plasticity (Alpino et al., 2024). Moreover, therapies aimed at SCFA receptors, specifically FFAR2/FFAR3, diminish exercise-induced enhancements in metabolic coupling and anxiety-like behaviors, so affirming that SCFA sensing is a pivotal mechanism governing the neurobehavioral impacts of physical activity (Lan et al., 2024).

At the peripheral-central integration level, SCFAs coordinate multi-loop communication. In the gastrointestinal tract, they prompt enteroendocrine cells to secrete hormones such as GLP-1, PYY, and serotonin, which engage vagal afferent pathways to influence limbic-hypothalamic networks associated with emotional regulation (Décarie-Spain et al., 2024). SCFAs can simultaneously traverse the blood-brain barrier by monocarboxylate transporters (e.g., MCT1), directly engaging in cerebral energy metabolism and neuronal signaling (Silva et al., 2020). This dual effect is facilitated by exercise-induced modifications in the gut-bile acid-intestinal peptide axis, which optimizes the spatiotemporal dynamics of SCFA signaling, hence augmenting its influence on brain circuits and behavior (Hsu and Schnabl, 2023).

At the molecular level, SCFAs significantly impact neuroplasticity and immunological equilibrium. SCFAs like as butyrate function as endogenous histone deacetylase (HDAC) inhibitors, augmenting histone acetylation (e.g., H3K9ac, H3K27ac) in brain areas including the hippocampus, hence facilitating the transcription of essential plasticity-related genes like Bdnf (Liu et al., 2018; Tran and Mohajeri, 2021). This epigenetic regulation collaborates with exercise-induced neurotrophic pathways to enhance synapse remodeling. Additionally, SCFAs contribute to the preservation of central immunological equilibrium by fostering a homeostatic microglial phenotype, suppressing pro-inflammatory signaling (e.g., NF-κB), and enhancing mitochondrial function (Colombo et al., 2021). They enhance the blood-brain barrier by upregulating tight junction proteins, so establishing a stable milieu favorable for cognitive function (Parker et al., 2020).

Studies on fecal microbiota transplantation further reinforce this causal relationship. Transferring gut microbiota from physically active donors to inactive recipients can reproduce the heightened SCFA levels and enhanced behavioral results seen in the donors, indicating that the exercise-induced microecological phenotype is transferable (Li et al., 2023). This evidence collectively supports a multi-pathway concept in which exercise increases SCFA availability, subsequently integrating signals at epithelial, endocrine, immunological, and brain interfaces. This mechanism concurrently enhances neuroplasticity via the vagus nerve, direct cerebral signaling, and epigenetic modulation, thereby augmenting emotional stability and cognitive efficacy (Cryan et al., 2020). Future investigations employing sophisticated methodologies like as stable isotope tracing and multi-omics integration will enhance our comprehension of the “exercise-SCFA-neuroplasticity” axis (Olateju et al., 2023).

### 4.4 Differences and commonalities among different exercise modes

Various exercise methods consistently enhance a beneficial axis of “SCFA production-barrier homeostasis-behavioral benefit,” however, the effect size and dynamics differ based on intensity, volume, and periodization (Min et al., 2024). Aerobic endurance training consistently improves SCFA-producing taxa and luminal SCFA concentrations, resulting in a fortified intestinal barrier, decreased inflammation, and enhanced mood and memory (Wang G. et al., 2021). This is accomplished by maximizing substrate accessibility, fostering a conducive gut environment, and improving SCFA transit and delivery (Duan et al., 2024). Resistance training, however, less researched, demonstrates potential by elevating acetate and propionate levels, which may synergize with myogenic signals to enhance central nervous system plasticity and emotional stability (Wagner et al., 2024; Lustgarten, 2019). When appropriately periodized with sufficient recovery, high-intensity interval training (HIIT) can enhance microbial diversity and SCFA levels, providing distinct benefits in metabolic flexibility (Wang et al., 2020).

However, to fully comprehend the heterogeneity of these outcomes, a critical analysis of confounding factors and physiological thresholds intrinsic to each modality is necessary. In the context of resistance training, the near-ubiquitous increase in dietary protein intake among practitioners serves as a major confounder. If not carefully counterbalanced by adequate fermentable fiber, a high-protein diet shifts distal colonic metabolism toward proteolytic, putrefactive pathways. This putrefaction generates branched-chain fatty acids (BCFAs) and potentially deleterious microbial metabolites, such as p-cresol and ammonia. The accumulation of these toxins can compromise intestinal epithelial cell metabolism and provoke local inflammation, thereby fundamentally negating the exercise-induced benefits on SCFA profiles and barrier integrity (Jäger et al., 2020). Similarly, understanding the concept of “maladaptation” is crucial for elucidating the “U-shaped” dose-response curve associated with HIIT and exhaustive endurance exercise. The specific thresholds that shift HIIT from beneficial to detrimental are heavily dependent on extreme intensities (e.g., prolonged efforts exceeding 80–90% VO2max) combined with inadequate recovery periods. During such intense physical stress, the physiological redistribution of cardiac output toward working skeletal muscles leads to a profound reduction in splanchnic blood flow, precipitating acute intestinal ischemia. The subsequent reperfusion phase generates excessive reactive oxygen species (ROS), which directly oxidative-stress the intestinal epithelium. This cascade provokes a temporary breakdown of tight junction proteins (e.g., claudins and occludin), resulting in exercise-induced gastrointestinal syndrome (EIGS) characterized by elevated permeability and systemic endotoxemia (Tomkovich and Jobin, 2016). Consequently, without gradual adaptation and adequate dietary fiber support, these severe ischemic events can completely overshadow the microecological benefits of training. The reaction to exercise is influenced by individual factors such as gender, age, and baseline microbiota, underscoring the necessity for individualized strategies to optimize the health advantages of the microbiota-SCFA pathway (Zhernakova et al., 2024).

Future research must implement stringent methodological requirements to guarantee strong and similar outcomes (Scheiman et al., 2019). This include the utilization of standardized diets (e.g., pair-feeding), regulation of training intensity and timing, and the application of thorough analytical methods for SCFA quantification (e.g., differentiating free and bound forms, incorporating portal vein measurements) (Zhang et al., 2025). Controlling for confounding variables such as stress is essential, particularly in animal models employing forced swimming (De Palma et al., 2014). Well-powered, stratified randomized controlled trials in people are necessary to elucidate the distinct benefits and synergistic potential of various exercise modalities (Dalile et al., 2019). This standardized, mechanism-driven approach will offer clear, evidence-based instructions for utilizing exercise to enhance physical and mental wellbeing via the gut microbiome.

## 5 Translational evidence in humans: associations and interventions

### 5.1 Cross-sectional associations: differences in fitness levels, exercise habits, and microbiota/SCFA profiles

Having established the fundamental molecular and cellular mechanisms, such as specific receptor activation, epigenetic modulation, and cross-feeding pathways, primarily through highly controlled animal models in section 4, this section shifts the focus to translational evidence in humans.

Cross-sectional studies consistently link elevated physical fitness and frequent exercise to a more diversified and stable gut microbiota, richer in SCFA-producing functional groups (Lavilla-Lerma et al., 2023). Taxa like Akkermansia and Bifidobacterium, which participate in mucus usage and cross-feeding, are frequently upregulated, associating with enhanced gut barrier function and reduced inflammation (Lin et al., 2025). Phenotypically, there are positive associations between VO_2_max or activity levels and fecal SCFA concentrations (Merghani et al., 2016), while circulating SCFAs are positively linked with heart rate variability (HRV) and decreased inflammatory markers (Kang and Wang, 2024). Interpreting circulating SCFA levels necessitates caution due to their significant dependence on portal absorption, hepatic first-pass metabolism, and peripheral clearance, resulting in conclusions that are less consistent than those derived from fecal data (Wachsmuth et al., 2022). Dietary and training attributes may influence specific “enterotypes,” exemplified by a Prevotella-dominant profile in high-fiber endurance athletes, potentially affecting individual exercise responses (Qin et al., 2022).

Regular exercisers demonstrate elevated gene clusters for SCFA synthesis and improved metabolic cross-feeding networks, wherein acetate generated by Bifidobacterium supports butyrate-producing bacteria (Abu-Ali et al., 2018). A significant “muscle-gut” metabolic connection is apparent, since exercise-induced lactate can be employed by intestinal bacteria to generate propionate (Scheiman et al., 2019). In addition to SCFAs, exercise is linked to modified profiles of secondary bile acids and other microbial metabolites, which collectively affect host physiology via multiple receptor-mediated and neurological pathways (Arab et al., 2020). This complex metabolic alteration highlights the comprehensive effects of exercise-induced microecological changes, connecting gut function to cardiovascular, metabolic, and immunological balance (Table 4).

**TABLE 4:** Gut microbiota and SCFA profiles associated with regular exercise/high fitness.

Association dimension	Key features	Key microbial taxa/pathways	Potential biological and physiological significance
Community structure and diversity	Increased α-diversity (Lavilla-Lerma et al., 2023). Distinct β-diversity signatures. Enhanced community stability and resilience.	Community-level metrics	Heightened functional redundancy. Improved ecosystem buffering against perturbations (e.g., diet, stress).
Abundance of key functional guilds	Enrichment of core butyrate-producing genera (Lin et al., 2025). Increased mucin-degrading and cross-feeding taxa.	Butyrate producers: *Roseburia, Faecalibacterium, Anaerostipes, Butyricicoccus*. Mucin degraders/Cross-feeders: *Akkermansia, Bifidobacterium*.	Strengthened gut barrier integrity. Attenuated local inflammation. Optimized microenvironment for efficient SCFA production.
Fecal and circulating SCFA levels	Fecal acetate, propionate, and butyrate positively correlate with exercise volume/VO*2*max. Favorable associations between circulating SCFAs and health markers (e.g., HRV) (Kang and Wang, 2024).	Major SCFAs: acetate, propionate, butyrate.	Reflects enhanced intestinal fermentation capacity. Suggests SCFAs mediate systemic benefits of exercise on metabolism and autonomic function.
Metabolic function pathways	Upregulation of SCFA synthesis gene clusters (Abu-Ali et al., 2018). Reinforced acetate-to-butyrate cross-feeding. Activation of lactate-SCFA axis via lactate-utilizing bacteria (Scheiman et al., 2019).	Pathways: Butyrate and propionate synthesis. Substrates: Lactate, acetate.	Improved carbohydrate fermentation efficiency. Host-microbe metabolic coupling via recycling of exercise-derived lactate into SCFAs.
Individual variation and enterotypes	Enterotype composition influenced by diet-exercise interactions (e.g., fiber/endurance vs. fat/strength) (Qin et al., 2022).	*Prevotella*-enterotype, *Bacteroides*-enterotype.	Baseline enterotype may determine the magnitude and nature of microbial response to exercise, explaining inter-individual heterogeneity.
Other metabolite profiles	Synergistic alterations in secondary bile acids, indole derivatives, and polyphenol metabolites (Arab et al., 2020).	Signaling molecules: Secondary bile acids, Indoles. Receptors: FFAR2/3, GPR109A, nuclear receptors.	SCFAs and other microbial metabolites form a complex signaling network, collectively modulating host cardiovascular, metabolic, and immune functions.

Notwithstanding these robust correlations, cross-sectional research is prone to considerable confounding factors. The diet serves as a principal confounder; sufficient fiber consumption enhances SCFA production, whereas high-protein and high-fat diets may negate the advantages of exercise (Hughes and Holscher, 2021). Additional factors, such as overall calorie consumption, body composition, age, pharmacological interventions, and circadian rhythms, systematically affect the microbiome (Cryan et al., 2019). Methodologically, it is essential to acknowledge that fecal SCFA concentrations indicate the unabsorbed residual rather than total production, and their interpretation must consider intestinal transit time and fecal water content (Mukhopadhya and Louis, 2025). Consequently, regulating these variables is crucial for precise interpretation.

Methodological uniformity is essential to enhance the evidence base. This entails employing objective metrics for physical activity (e.g., accelerometers), comprehensive dietary documentation, and absolute quantification of microbial abundance to address the shortcomings of relative data (Clauss et al., 2021). Standardized sample handling and the reporting of absolute concentrations are essential for metabolomics (Duan et al., 2024). Future research should include longitudinal and intervention studies, such as randomized controlled trials, using stringent dietary controls (isocaloric/isofiber) and multi-omics integration. By concurrently assessing endpoints such as barrier function and heart rate variability, while stratifying analyses by significant variables, the field can transition from strong ecological correlations to reproducible, causal findings, thereby reinforcing the significance of the “exercise-microbiota-SCFA” axis in public health (Wong et al., 2025).

### 5.2 Interventions studies: effects of aerobic and resistance training on human gut microbiota and SCFAs

Intervention studies offer strong evidence that moderate, regular exercise enhances SCFA-related ecological and metabolic processes, influenced by training modality, dosage, and individual characteristics (Chimera et al., 2025). Moderate-intensity aerobic training regimens (e.g., 6–12 weeks) reliably augment the gut’s capacity for SCFA production, frequently resulting in elevated fecal butyrate and propionate levels (Hintikka et al., 2023). This effect is correlated with improvements in cardiorespiratory fitness (VO_2_max) and dietary fiber consumption in a dose-dependent manner (Wang R. et al., 2021). Importantly, these adaptations can be reversed with cessation of training, underscoring the importance of continuous exercise for enduring advantages (Cronin et al., 2018). Although training might independently contribute to certain benefits, food habits can either enhance or obscure these effects (Chimera et al., 2025). Simultaneously, certain studies indicate enhancements in host barrier integrity and reduced low-grade inflammation; nonetheless, these findings necessitate additional validation through larger, methodologically consistent trials (Chen et al., 2024).

Aerobic training consistently enhances gene modules associated with carbohydrate fermentation and butyrate production, especially the butyryl-CoA: acetate CoA-transferase route (Mukhopadhya and Louis, 2025). This is frequently associated with increased activity in the cross-feeding network between acetate-producing Bifidobacterium and butyrate-producing commensals (Canfora et al., 2022). Moreover, endurance training may enhance a “muscle-gut” metabolic connection by augmenting the ability of certain bacteria to transform exercise-induced lactate into propionate (Scheiman et al., 2019). It is crucial to recognize that whereas fecal SCFA levels exhibit generally stable alterations, the associated signals in peripheral circulation tend to be weaker and more variable due to the intricate interactions of intestinal absorption, hepatic first-pass metabolism, and peripheral tissue clearance (Mukhopadhya and Louis, 2025).

Emerging evidence indicates that resistance training and HIIT exhibit considerable variation (Mailing et al., 2019). Progressive resistance training, especially in at-risk groups, correlates with small increases in butyrate-producing bacteria and total SCFAs, which coincide with enhancements in muscle strength and insulin sensitivity (Aya et al., 2023). Nevertheless, these programs are sometimes complicated by elevated protein consumption, which may induce protein fermentation if not well counterbalanced with sufficient dietary fiber (Ross et al., 2024). HIIT can enhance microbial diversity and SCFA activities, provided it is appropriately periodized with sufficient recovery; maladaptation may result in temporary barrier stress (Wang et al., 2020). This highlights the essential equilibrium between training load and recovery in influencing the overall microecological result.

The extent of exercise-induced alterations is determined by various factors. The FITT principles (frequency, intensity, duration, type) are fundamental determinants, although baseline parameters such as age, fitness level, and gut microbiota composition influence individual response trajectories (Mailing et al., 2019). Methodologically, interpreting data necessitates consideration of factors such as intestinal transit time, as fecal SCFAs indicate unabsorbed “residual” amounts rather than total production (Mukhopadhya and Louis, 2025). Future research must utilize rigorous methodologies, including randomized controlled trials with isocaloric and isofibrous dietary controls, to progress the area (Chimera et al., 2025). Integrating multi-omics analyses with objective assessments of training adherence and physiological adaptation is crucial for translating existing findings into solid, evidence-based guidelines for enhancing metabolic health through exercise (Muller et al., 2024).

### 5.3 Studies measuring both microbiota/SCFA and psychological/cognitive outcomes: preliminary human evidence for the association chain

Interventional studies that simultaneously assess microbiota/SCFA and psychological or cognitive outcomes typically demonstrate a consistent positive trend: following regular aerobic or combined training, the reduction in depression, anxiety, and perceived stress scales is frequently associated with a relative increase in butyrate-producing bacteria and elevated levels of butyrate and propionate in feces or circulation (Zeng Q. et al., 2025; Riezzo et al., 2023); certain studies also document enhancements in executive function, working memory, and learning task performance, which coincide with the upregulation of SCFA-producing functional modules, beneficial alterations in low-grade inflammation, and intestinal barrier markers (Kang and Wang, 2024). Certain trials employing statistical mediation models indicated that variations in SCFA partially elucidate the indirect effects of exercise on mood or stress indicators (Loh et al., 2024). Concurrent studies assessing autonomic nervous function revealed that the enhancement of HRV and sleep quality aligned with the increased capacity for SCFA production, implying a potential coordinated involvement of the “gut-brain axis” and “heart-gut-brain” pathways (Dalton et al., 2019).

**FIGURE 4:**
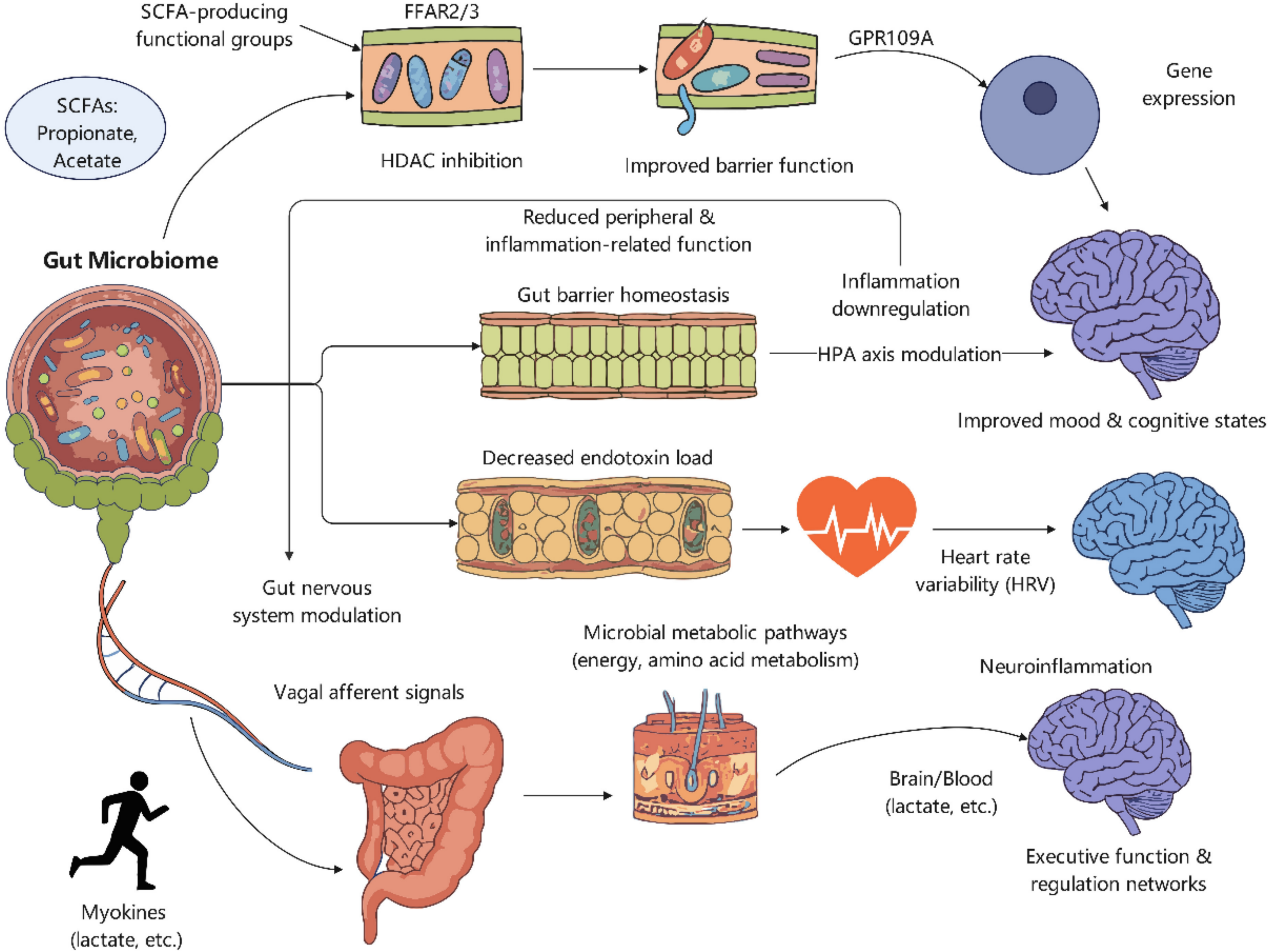
Hypothesis model of multi-pathways through which exercise promotes mental and cognitive health mediated by SCFAs. This figure illustrates how exercise-induced SCFA gains provide mechanistic rationales for brain health through four parallel and interrelated biological pathways: (1) Endocrine signaling via gut peptide release; (2) Neural communication via vagal afferent activation; (3) Systemic immune regulation through the induction of regulatory T cells and suppression of pro-inflammatory cytokines; and (4) Direct epigenetic modulation of neurotrophic factors in the brain. Together, these convergent pathways alleviate depressive symptoms, buffer the HPA axis stress response, and optimize executive function. Figure was created with EdrawMax.

Translating the molecular mechanisms detailed in section 4 to human physiology, the systemic effects of SCFAs on mental and cognitive health are increasingly supported by clinical biomarkers rather than direct receptor assays. Instead of reiterating specific epigenetic or receptor-mediated pathways, it is crucial to emphasize that in human trials, exercise-induced SCFA increases correlate directly with quantifiable reductions in systemic inflammatory cytokines and improvements in circulating markers of barrier integrity (Chen et al., 2024). Furthermore, the enhancement of SCFA-producing functional groups frequently correlates with a reduction in serum endotoxin levels, which macroscopically supports the hypothesis of improved mood and cognitive function via the down-regulation of the HPA axis stress response in human subjects (Cryan et al., 2020; Figure 4). Additionally, the modulation of the autonomic nervous system by the gut microbiome provides a physiological foundation for the identified statistical mediation between clinical HRV improvements and mood enhancement (Bonaz et al., 2018). Evidence from human metabolomic profiling indicates that exercise-induced myogenic signals intersect with microbial metabolic pathways, which may indirectly influence clinical executive function networks by affecting the systemic tryptophan-kynurenine ratio (Brown et al., 2021).

It is essential to highlight that existing human evidence for the “exercise-microbiota/SCFA-brain” axis is primarily correlational and has substantial constraints (Kang and Wang, 2024). Many studies are marked by limited sample sizes, brief intervention periods, and other confounding variables, such as simultaneous dietary modifications, weight reduction, and enhanced sleep, which might conceal the distinct benefits of the microbiome pathway (Rohmann et al., 2024). Methodological problems remain; fecal SCFA levels indicate unabsorbed residues rather than total synthesis, whereas circulating levels are inconsistently affected by hepatic metabolism (Dalile et al., 2019). Moreover, dependence on self-report measures for mental health outcomes may induce placebo effects, while even objective neurobehavioral assessments are susceptible to practice effects (Aucoin et al., 2020).

Future studies must employ more stringent designs to enhance causal inference (Kang and Wang, 2024). This entails executing randomized controlled trials with active controls, standardized isocaloric and isofibrous diets, and bigger, stratified cohorts to augment statistical power and generalizability (Palmer et al., 2022). A multi-timepoint measurement approach is crucial, incorporating multi-omics data with objective physiological indicators such as inflammatory panels, heart rate variability, and sleep measures, in conjunction with validated cognitive assessments (Muller et al., 2024). Pre-registered mediation and time-lagged models ought to be utilized to measure the precise impact of SCFAs on exercise-induced enhancements in mood and cognition, as well as to investigate possible dose-response associations (Kang and Wang, 2024).

In summary, whereas current intervention studies offer initial evidence indicating that regular exercise enhances mental health through the microbiota-SCFA route, establishing these correlations as robust causal proof necessitates considerable methodological improvements (Mailing et al., 2019). An integrated approach that merges an active lifestyle with a nutritious food, sufficient sleep, and effective stress management is likely to optimize the positive modulation of the gut-brain axis (Xiong et al., 2023). By conducting more precise, multidisciplinary research, we may thoroughly clarify this route and convert the findings into practical public health recommendations for enhancing holistic wellbeing (Wu et al., 2021).

### 5.4 Critical discussion: key considerations on confounding, measurement, and design

Current human research examining the “exercise-microbiota/SCFA-mental health” relationship is primarily associative. While preclinical models strongly suggest that SCFAs mediate the benefits of exercise, human data remain largely correlational, and any definitive causal inference requires further validation. These studies are hindered by systematic limitations that undermine the credibility of causal conclusions. A primary concern is the insufficient regulation of confounding variables, especially nutrition and lifestyle (Rohmann et al., 2024). Many studies inadequately regulate or accurately document dietary intake of fiber, polyphenols, and total energy, complicating the differentiation of exercise effects from dietary modifications (Mailing et al., 2019). Critically, specific and potent confounders are frequently overlooked. The pervasive use of medications, including proton pump inhibitors (PPIs), metformin, antibiotics, and even over-the-counter non-steroidal anti-inflammatory drugs (NSAIDs), can profoundly alter the gut microbiome and SCFA profiles, yet these are rarely controlled for in exercise trials. Furthermore, while general sleep is occasionally noted, it must be intricately linked to circadian biology; circadian disruptions driven by shift work, poor sleep architecture, and irregular meal timing are known to severely dysregulate microbial homeostasis. Alongside stress and alcohol intake, the failure to measure these variables introduces significant noise into the data (Smith et al., 2019). Moreover, exercise prescriptions frequently lack consistency, exhibiting varied adherence and a discrepancy between intended and actual training loads, so complicating the analysis of dose-response correlations (Cronin et al., 2018).

A significant barrier resides in the design of the study and the characteristics of the sample. Numerous research are hindered by limited sample sizes, which lack the statistical power necessary to identify minor effects or perform significant subgroup analysis (Kang and Wang, 2024). The variability in participant characteristics, including gender, age, BMI, and metabolic status, is frequently insufficiently addressed by stratification, hence constraining the generalizability of the results (Kang and Wang, 2024). Power-based sample size estimation, multi-center recruiting, and pre-defined analyses of varied populations are essential for improving the external validity and rigor of forthcoming research (Rohmann et al., 2024).

Ultimately, the lack of methodological consistency in measuring is severely inadequate, compromising data quality and the comparability of studies (Knight et al., 2018). Fecal SCFA assays exhibit significant sensitivity to factors such as sampling duration, intestinal transit, and sample pre-treatment, but circulatory SCFA levels are minimal and readily affected by fasting status (Dalile et al., 2019). A transition from 16S rRNA sequencing to metagenomics with absolute quantification is essential for enhanced functional understanding at the microbiological level (Barlow et al., 2020). To enhance causal inference, forthcoming studies should implement robust methodologies, including randomized controlled trials with pre-registered mediation models that consider competing mediators (e.g., alterations in fitness, inflammation) (Loh et al., 2024). Implementing these enhancements—such as supervised training, objective monitoring through wearables, and standardized multi-omics protocols—will enable the discipline to provide more reproducible findings to inform translational applications in advancing holistic health (Muller et al., 2024).

## 6 The critical moderator: dietary fiber and exercise synergy

**FIGURE 5:**
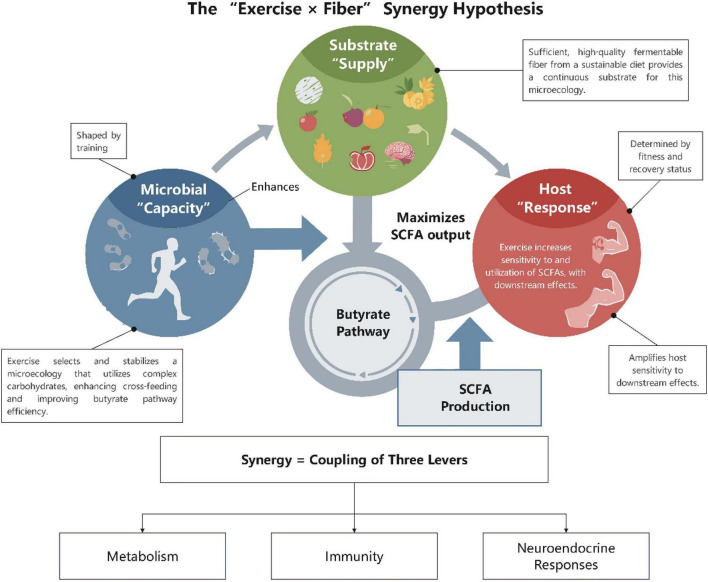
The “Exercise × Dietary Fiber” synergistic gain hypothesis: Maximizing SCFA-Mediated Health Effects. Regular exercise and adequate intake of high-quality fermentable dietary fiber work synergistically to form a positive feedback loop. This synergy operates across three interdependent domains: Microbial Capacity: Exercise primes the intestinal niche (e.g., via mucus turnover and optimized redox state) to favor SCFA-producing bacteria and cross-feeding networks. Substrate Supply: Dietary fiber provides the essential continuous raw materials for saccharolytic fermentation. Host Response: Exercise upregulates SCFA transporters (e.g., MCT1) and enhances peripheral tissue sensitivity. This tripartite coupling maximizes the overall production, absorption, and biological efficacy of SCFAs. Figure was created with EdrawMax.

Evidence from many human and prospective animal research indicates that exercise and food collaboratively influence the gut microbiota and its synthesis of SCFAs at multiple levels (Song et al., 2022). Consistent exercise enhances the microbiota and functional pathways associated with SCFA production, increases epithelial transport and signaling mechanisms, and strengthens barrier and immune functions; however, in the absence of sufficient fermentable substrates, these adaptations may not result in elevated SCFA production (Yin et al., 2022). The “exercise × fiber” synergy hypothesis is postulated based on this. Exercise curates and stabilizes a microecology adept at metabolizing complex carbohydrates, enhances cross-feeding, and optimizes the butyrate pathway’s efficiency; concurrently, adequate and high-quality fermentable dietary fiber offers a persistent substrate for this microecology, thereby maximizing SCFA production (Xie and Huang, 2024); simultaneously, exercise augments the host’s sensitivity and utilization of SCFAs, intensifying downstream effects such as metabolism, immunity, and neuroendocrine responses (Chen et al., 2024). This synergy can be encapsulated as the synchronized interaction of three factors: microbial “capability” (influenced by training), substrate “availability” (dictated by the quantity and quality of fiber), and host “reaction” (governed by fitness and recovery status) (Song et al., 2022; Figure 5).

The nature and quality of fibers are critically significant. Soluble, highly fermentable fibers often enhance the synthesis of acetate and propionate while facilitating butyrate production via cross-feeding (Yang and Cong, 2021). Resistant starches (including RS2/RS3 from unripe green bananas, cooked and cooled potatoes or rice, legumes, oats, and barley) are consistently linked to increased fecal or circulatory butyrate levels (Li et al., 2024). A variety of whole-food fibers frequently includes synergistic components such as polyphenols, which can collaboratively enhance advantageous saccharolytic fermentation networks (Mailing et al., 2019). In contrast, high-protein and low-fiber diets may redirect distal colonic fermentation toward protein pathways, diminishing SCFA synthesis and elevating potentially detrimental metabolites (Danneskiold-Samsøe et al., 2019). Concerning dose, adults ought to incrementally elevate their overall fiber consumption to roughly 25–38 grams daily, emphasizing fermentable elements. Gradually increasing by 5–10 g weekly, coupled with sufficient water and electrolyte consumption, may alleviate gastrointestinal pain and improve adherence (Barber et al., 2020).

The time of consumption and physical activity also governs this synergy. Colon fermentation is affected by circadian rhythms and substrate arrival times; moderate-intensity exercise can modify intestinal peristalsis and mixing, thereby influencing the timing of SCFA presence in stools and circulation (Varghese et al., 2024). Practical advice entail: scheduling high-fermentable fibers 2–3 h prior to high-intensity or prolonged endurance training, and dispersing fiber-rich foods throughout the day to ensure a consistent supply of substrates; Mild aerobic or resistance training typically aligns well with regular meals containing fiber; incorporating fermentable fibers and resistant starch into the post-exercise recovery meal can enhance microbial recovery and SCFA synthesis during the ensuing rest period (Jäger et al., 2019). Individuals susceptible to exercise-induced gastrointestinal distress can adjust the kind and time of fiber according to personal tolerance, while preserving overall daily intake and consistent scheduling to regulate fermentation patterns and improve comfort (Koivisto-Mørk et al., 2020).

**FIGURE 6:**
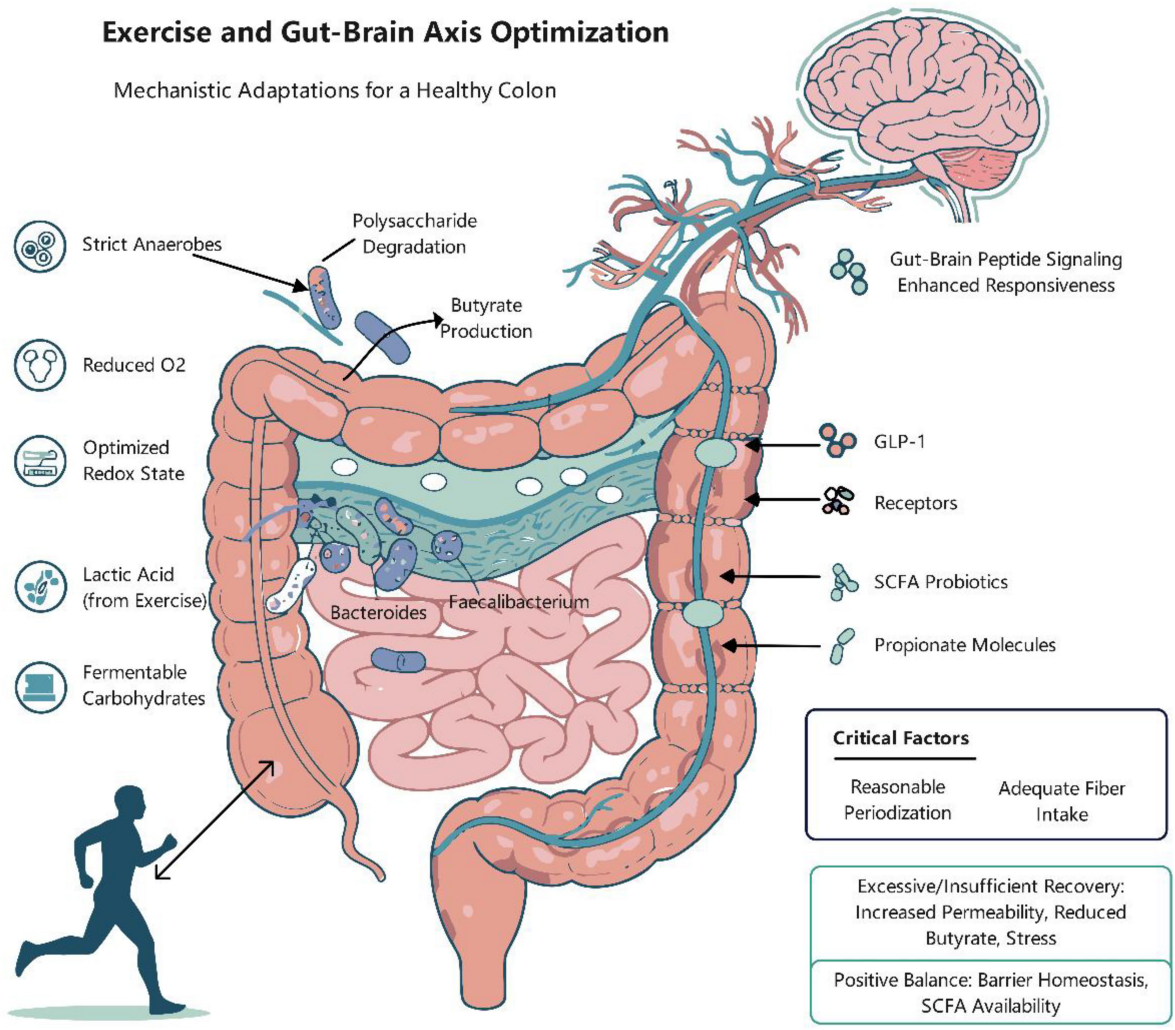
Adaptive regulatory mechanisms of regular exercise on gut microbiota and host responses. Regular and moderate exercise systematically optimizes the intestinal microenvironment through several key physiological adaptations. Exercise lowers luminal redox potential (favoring strict anaerobes), optimizes intestinal transit time (expanding the proximal fermentation window), and increases mucus turnover (providing endogenous substrates for mucin-degraders). Furthermore, exercise-induced adaptations enhance the host’s response by upregulating tight junction proteins to decrease permeability, and improving mesenteric microcirculation, thereby facilitating the efficient transport and systemic distribution of microbial metabolites, especially SCFAs. Figure was created with EdrawMax.

At the molecular level, the mechanistic explanation for how exercise primes the microbiota to more efficiently utilize fiber extends well beyond the broad concept of “creating an ecological niche.” Instead, this synergy is driven by specific, physiologically-grounded multi-level interactions. First, regarding the luminal redox state, regular exercise lowers the colonic redox potential, creating a highly reduced, hypoxic environment that strictly favors the proliferation of obligate anaerobic SCFA producers. Second, exercise dynamically regulates mucus turnover by mechanically and physiologically increasing intestinal mucus production. A thicker, frequently renewed mucus layer provides both a structured habitat and a slow-release endogenous substrate for mucin-degrading specialists, most notably *Akkermansia muciniphila*. The acetate produced by *Akkermansia* subsequently serves as a crucial primary substrate for cross-feeding butyrate producers (e.g., *Faecalibacterium and Roseburia*). This establishes a robust positive feedback loop: exercise provisions the structural niche and starter fuel, while exogenous dietary fiber sustains the broader saccharolytic network. Third, moderate exercise optimizes gut transit time. By inducing a slightly prolonged transit specifically in the proximal colon, exercise effectively expands the “fermentation window.” This temporal optimization allows for a more complete and efficient microbial breakdown of complex, highly polymerized fermentable fibers. In addition to these structural and temporal optimizations, increases SCFA transport and receptor signaling, and amplifies the sensitivity of gut-brain peptides to SCFA (Palmer et al., 2022; Figure 6). Training-induced microbiota alterations facilitate a cross-feeding network from primary polysaccharide degraders to butyrate producers; lactate produced during exercise can additionally serve as a substrate for certain propionate producers, thereby enhancing the integration of host metabolism and intestinal fermentation (Scheiman et al., 2019). These modifications are most evident under conditions of a continuous supply of fermentable carbohydrates and can diminish the pathological over-mobilization of mucin as an alternate substrate during dietary fiber deprivation, hence aiding in the maintenance of barrier homeostasis (Schroeder, 2019). Excessive or inadequate recovery training may transiently elevate intestinal permeability, diminish butyrate availability, and provoke short-term stress responses; appropriate periodization, sufficient sleep, and adequate fiber intake are beneficial for sustaining a favorable balance (Wang et al., 2020).

At the transformative level, implementing a factorial design of “exercise prescription × fiber dosage” while stratifying baseline dietary fiber consumption and the functional capacity of the microbiota is anticipated to optimize health outcomes associated with SCFAs in the community (Xie et al., 2024). Practical integrated solutions encompass: engaging in consistent and moderate aerobic and/or resistance training with sufficient recovery; emphasizing whole foods and varying the consumption of fermentable fibers, supplemented with specific prebiotics if required; adjusting the types and timing of fiber intake according to individual tolerance and training schedules; and enhancing the overall dietary framework to promote glycolytic fermentation while suppressing excessive protein fermentation (Song et al., 2022). The interplay between physical activity and dietary fiber is anticipated to strengthen microecological function, augment SCFA signaling, and reinforce multifaceted advantages in metabolism, immunity, and neurocognition (Liu et al., 2025). This strategy advocates for an active lifestyle and balanced nutrition, fostering a mutually beneficial outcome for personal health and societal cohesion.

## 7 Methodological considerations and future directions

Methodological rigor must be improved and standardized to further research on the gut-microbiota-brain axis. A major current limitation is the profound heterogeneity in how different types (e.g., aerobic versus resistance training), intensities (e.g., moderate versus high-intensity interval training), and durations of exercise differentially affect gut microbiota composition and SCFA production. To accurately delineate these specific effects and reduce inter-study variability, we strongly call for the explicit standardization of research practices across three key domains. First, exercise prescriptions must be rigorously standardized and reported using the FITT (Frequency, Intensity, Time, Type) principles, documenting both the prescribed load and actual adherence. Second, the timing of sample collection must be highly controlled; specifically, the concurrent collection of fecal and blood samples should be standardized to capture precise acute post-exercise windows versus chronic baseline adaptations, supported by meticulous documentation of factors such as intestinal transit time and fecal pH (Vandeputte et al., 2016). Establishing pre-registered standard operating procedures for sample handling, encompassing time-stamping and cold-chain management, is essential to mitigate batch impacts. For SCFA analysis, GC-MS/LC-MS utilizing stable isotope internal standards is the chosen methodology, guaranteeing precise quantification and clear reporting of analytical performance. Microbiota analysis should advance from 16S rRNA sequencing to metagenomics for precise quantification and functional pathway annotation (Barlow et al., 2020). Psychological and exercise-related outcomes must be standardized, documenting both prescribed and actual training loads while employing proven, objective measurements to mitigate confounding effects.

A comprehensive future research program must include stringent experimental designs to evaluate causal theories. The current “Exercise × Fiber Synergy” theoretical framework relies largely on inferential synthesis from independent exercise or dietary cohorts. Direct empirical evidence concurrently testing this synergistic interaction on mood and cognitive outcomes remains lacking in the literature. To definitively bridge this gap, future research must prioritize the 2 × 2 factorial design (e.g., exercise vs. no exercise × high-fiber vs. low-fiber diet), under isocaloric settings, which is optimal for specifically examining the interaction between exercise and dietary substrates on SCFA kinetics and subsequent mental health outcomes (Zeng L. et al., 2025). Employing randomized controlled or crossover designs with pre-registered mediation analyses grounded in a counterfactual paradigm will facilitate a more accurate assessment of the mediating function of SCFAs. To enhance the validation of causality, “instrumental” interventions may be utilized, such the application of specific prebiotics to adjust substrate availability, targeting SCFA receptor pathways, or, with stringent ethical supervision, performing fecal microbiota transplantation from physically active donors. These methodologies, coupled with *in vitro* fermentation models and stable isotope tracing, will furnish mechanistic validation for *in vivo* observations (Agus et al., 2021).

Ultimately, research should concentrate on accuracy and population-level implementation to ascertain “who derives the greatest benefit.” From a translational perspective, this synergistic theory offers profound clinical and public health applications. Rather than relying on singular interventions, practitioners could formulate “combined lifestyle prescriptions,” strategically pairing specific exercise modalities with targeted fermentable fiber intake to maximize SCFA yield and synergistically alleviate symptoms of depression or cognitive decline. However, transitioning this theory into real-world practice faces notable implementation barriers. First, long-term dietary adherence to high-fiber regimens remains notoriously challenging in general populations. Second, baseline microbiome heterogeneity significantly dictates inter-individual variability; patients lacking foundational keystone species (e.g., Akkermansia or specific butyrate producers) may experience a blunted synergistic response. To overcome these barriers, future efforts must entail entails constructing prediction models that amalgamate baseline microbiome data with host attributes to facilitate personalized exercise and nutritional recommendations, which must subsequently be validated in independent cohorts (Zeevi et al., 2015). Prioritizing the expansion of research to clinical and geriatric populations via multi-center trials incorporating sustainable exercise programs is essential (Ni Lochlainn et al., 2024). These trials ought to include composite outcomes centered on cognition, physical fitness, and quality of life, while meticulously accounting for comorbidities and pharmacotherapy. By adhering to a principal framework of “standardized measurement + rigorous design + mechanism validation + individualized translation,” the discipline can produce high-quality, replicable data to efficiently incorporate exercise and nutrition into public health initiatives aimed at enhancing holistic wellbeing.

## 8 Conclusion

Current data indicates that regular exercise enhances brain health and psychological wellbeing by enhancing the functional composition and metabolic pathways of gut microbiota, augmenting the production and communication of SCFAs, and facilitating multi-system coordination. This mechanistic framework is both biologically viable and practically implementable: training can enhance fiber utilization and cross-substrate networks, while the host upregulates SCFA transport and receptor sensitivity, ultimately yielding beneficial effects on mood, cognition, and stress recovery. This conclusion remains a “strong hypothesis” at this time. Despite corroboration from several sources in both animals and humans, there remains a necessity for high-quality, standardized human causal data to further validate the effect magnitude, dose-response relationship, and applicable parameters.

Furthermore, this is not a singular channel but rather enhances the traditional advantages of physical activity. Neurotrophic factors, including BDNF, the augmentation of synaptic plasticity, the enhancement of cardiopulmonary and cerebral hemodynamics, the optimization of glucose and lipid metabolism, the reconfiguration of sleep quality and circadian rhythms, along with the down-regulation of systemic inflammation and oxidative stress, are anticipated to synergistically interact with SCFA-mediated gut-brain signaling, collectively establishing a “multi-node, weakly coupled yet robust” health network. In this network, dietary fiber serves as a crucial substrate that interacts with training adaptation, simultaneously enhancing the microbiota’s “capacity” and the host’s “response,” thus converting individual regular exercise and a balanced diet into a cohesive and sustainable advantage for both physical and mental wellbeing.

In practice, developing exercise recommendations must incorporate an individual’s baseline microbiome traits and dietary patterns, personalizing the optimization concerning exercise modality, intensity, frequency, and meal timing, while also ensuring an adequate quantity and type of fermentable dietary fiber and resistant starch to promote the production and utilization of SCFA. The execution of prescriptions can be flexibly modified according to basic physiological and behavioral metrics, emphasizing sustainability and safety. Despite existing evidence gaps regarding population heterogeneity, long-term follow-up, causal pathways, and measurement standardization, the potential for translational application appears promising due to advancements in factorial randomized controlled trials, mediation analysis, and instrumental interventions, alongside enhanced data sharing and interdisciplinary collaboration. Promoting the integration of vigorous activity with a high-fiber, whole-food diet is anticipated to enhance individual mental health and healthy aging, while also cultivating a good, mutually supportive, and harmonious social environment.
